# Slow evolution of Europa’s interior: metamorphic ocean origin, delayed metallic core formation, and limited seafloor volcanism

**DOI:** 10.1126/sciadv.adf3955

**Published:** 2023-06-16

**Authors:** Kevin T. Trinh, Carver J. Bierson, Joseph G. O’Rourke

**Affiliations:** School of Earth and Space Exploration, Arizona State University, AZ 85287, USA.

## Abstract

Europa’s ocean lies atop an interior made of metal and silicates. On the basis of gravity data from the Galileo mission, many argued that Europa’s interior, like Earth, is differentiated into a metallic core and a mantle composed of anhydrous silicates. Some studies further assumed that Europa differentiated while (or soon after) it accreted, also like Earth. However, Europa probably formed at much colder temperatures, meaning that Europa plausibly ended accretion as a mixture containing water-ice and/or hydrated silicates. Here, we use numerical models to describe the thermal evolution of Europa’s interior assuming low initial temperatures (~200 to 300 kelvin). We find that silicate dehydration can produce Europa’s current ocean and icy shell. Rocks below the seafloor may remain cool and hydrated today. Europa’s metallic core, if it exists, may have formed billions of years after accretion. Ultimately, we expect the chemistry of Europa’s ocean to reflect protracted heating of the interior.

## Teaser

Unlike Earth, Europa may have differentiated slowly and spent most or all of its life without a fully formed metallic core.

## INTRODUCTION

One of the best candidates for habitability in our solar system is Jupiter’s moon, Europa (*1*–*3*). A global salt water ocean churns between the rocky mantle and ice shell (*4*–*12*). Many studies argued that rock-water reactions can deliver biologically vital elements and energy across Europa’s seafloor (*1*–*3*, *13*, *14*). One proposed mechanism to sustain ocean habitability is serpentinization: the hydrous alteration of ultramafic rock. Serpentinization is a ubiquitous process on Earth in both hot (e.g., hydrothermal vents) (*15*) and cold (e.g., continental subsurface) (*16*) systems. Rock-water interactions in many planetary contexts are conducive to serpentinization, as supported by detection of serpentine minerals in carbonaceous chondrites (*17*–*19*) and the Martian surface (*20*, *21*). In addition, volcanism may contribute volatiles to the ocean if silicates melt near the seafloor (*22*, *23*), thus driving chemical disequilibria in the ocean that is necessary for life as we know it. If and when these processes happen at Europa depends on the evolving physical conditions of the rock-metal interior.

Europa’s ocean composition depends on, among other factors, the temperature and pressure conditions where water meets rock. Roughly speaking, there are two regimes of plausible rock-water reactions at Europa: near the seafloor and in the deep interior. In the first case, reactions happen at the seafloor or within the top tens of kilometers of silicates. Serpentinization may occur at ambient temperatures (*16*) or, in the case of Earth-like temperature hydrothermal vent systems, up to ~670 K (*13*). Geochemical models that focus on such low to moderately high temperatures predict a sulfate-rich ocean at Europa (*24*–*27*), assuming that reactions proceed to chemical equilibrium (i.e., not kinetically inhibited) (*28*). Some have argued that a sulfate-rich ocean explains the detection of sulfates on the trailing side of Europa (*29*–*32*), although radiolytic chemistry of oxidants delivered from Io could also produce sulfates on Europa’s surface (*33*–*35*). On the other hand, Europa could dehydrate its deep interior silicates at ~550 to 900 K and a few gigapascals to produce multicomponent fluids (*36*). Some groups speculated that hydrated silicates, rather than accreted ice, may be the source for Europa’s ocean and ice shell (*24*, *37*, *38*). A metamorphic origin may result in an ocean that is rich in Cl compared to S at shallow depths (*38*), which could explain the recent detection of potentially endogenous sodium chloride on the surface of Europa (*39*–*41*). However, ocean circulation and/or other processes may reduce the aforementioned Cl:S ratio. The extent to which surface material reflects ocean chemistry and composition depends on ocean and ice shell dynamics, which remain an ongoing topic of investigation (*12*, *42*).

The physics and chemistry of the rock-metal interior dictates Europa’s overall evolution. Rock and metal contribute >90% of Europa’s total mass (*4*, *7*–*11*, *43*). The prevailing view of Europa’s interior argues for four compositionally distinct layers: a metallic core, silicate mantle, subsurface ocean, and icy shell (*8*–*11*, *14*, *28*, *44*, *45*). The primary observational constraints on Europa’s internal structure are mass, radius, and normalized moment of inertia (MoI). Lower MoI values signify that mass is more concentrated toward the planetary center. Initial interpretations of radio Doppler (i.e., gravity) measurements from the *Galileo* mission inferred a low MoI value of 0.346 ± 0.005 from two gravity coefficients (J_2_ and C_22_) assuming hydrostatic equilibrium using the Radau-Darwin approximation (*4*). However, recent reanalysis of *Galileo* data found that one gravity coefficient (C_22_) is larger than previous estimates (*43*). This result entails that Europa’s MoI may be larger than previously assumed, thus the interior is less differentiated. The existence of a metallic core in Europa, while taken for granted by some prior studies, is again an open question.

Europa probably did not form a metallic core during its accretion (if the metallic core formed at all). Fe-FeS alloys melt at ~1250 to 1950 K from the eutectic point to pure iron at Europa’s central pressure (~4 GPa), respectively (*46*, *47*). However, Europa may have accreted cold. We can see this with a simplified calculation of temperature increase, driven by accretional heat: Δ*T_G_ = −*(*3hGM*)/(*5Rc*_p_). Here, Δ*T_G_* is the accretional warming, *h* is the heat retention, *G* is the gravitational constant, *M* is the total mass, *R* is the total radius, and *c*_p_ is the specific heat capacity. If we set *c*_p_ to a value typical of silicates (1000 J kg^−1^ K^−1^) and unrealistically assume the upper limit of *h* = 1, then accretion could warm up primordial Earth by ~38,000 K. For Europa’s mass and radius, the temperature increase would only be ~1200 K. In this improbably warm case, Europa may still not have enough gravitational energy to trigger metallic core formation. Planetary bodies tend to accrete slowly such that some accretional heat radiates back into space (i.e., *h* < 1). In the framework of this simple calculation, *h* ~ 0.1 would best match geochemical constraints on the thermal conditions of Earth’s accretion (*48*). The value of *h* for Europa is not known with certainty, although numerous models (e.g., the “gas-starved disc” model) suggest that *h* << 0.01 for the Galilean moons due to their long accretion time (*28*, *49*). Europa’s initial temperatures would match the circumjovian disc where it formed (~200 to 300 K), which are too cool to provoke metallic core formation (*28*) or even silicate dehydration. Therefore, unlike the larger rocky planets, Europa may have been too cold to form a metallic core until recently.

**Fig. 1.**
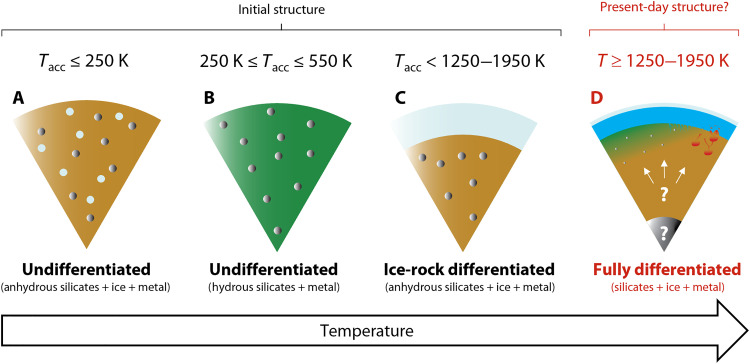
Unlike large rocky planets, Europa’s accretional temperatures were probably too low for metallic core formation. Instead, Europa likely formed as a rock-metal undifferentiated body [between endmembers (**A** to **C**)] and experienced slow structural changes driven by radiogenic and potentially tidal heating. The age of Europa’s metallic core is unknown. However, Europa could be ice-rock-metal differentiated today (*4*, *7*–*11*, *43*) as shown by endmember (**D**). Previous studies have modeled Europa’s evolution assuming endmember B (*24*, *38*), endmember C (*28*, *44*), and endmember D (*45*, *62*, *67*) as initial conditions. However, endmember D is unlikely because Europa would need to accrete fast (i.e., retain all accretional heat) and have a metallic core composition near the Fe-FeS eutectic (~24 to 28 wt % S), which melts at ~1250 K (*46*). Numerous formation models argue that Europa formed at low temperatures where water-ice may exist (*49*, *80*, *81*, *89*). The brown and green colors represent anhydrous and hydrous silicates, respectively. Light blue represents liquid water and/or ice. Silver represents metal. The degree to which primordial Europa is dehydrated and differentiated depends on the composition of the satellite’s accreted material, initial temperatures, *T*_acc_, and subsequent heat production.

We explore three cases that serve as end-member examples of Europa’s initial state after its accretion (Fig. 1). In one case, Europa could accrete as a mixture of anhydrous rock, metal, and ice (Fig. 1A). The ocean would form first when the accreted ice melts. Radiogenic and tidal heating would then warm up the interior and cause ice-rock and rock-metal differentiation at ~250 to 273 K (*50*) and ~1250 to 1950 K (*46*), respectively. Alternatively, Europa could instead form mostly from hydrous silicates and metal (Fig. 1B). At ~550 to 900 K, the hydrated silicates would release chemically bonded H and OH as water (*36*). This metamorphic fluid would mostly be supercritical and rich in aqueous solutes derived from host rock (*38*). Some groups proposed that Europa formed its ocean-ice shell by silicate dehydration (*24*, *25*, *37*, *38*, *51*) before reaching the high temperatures necessary for metallic core formation. Last, Europa could form as a bulk mixture of anhydrous rock and metal, covered by an ice shell (Fig. 1C). This endmember scenario resembles the one in Fig. 1A but implies that ice-rock differentiation occurred during accretion. Starting from any of these cases (or some intermediate state), Europa then experienced substantial structural changes before reaching its present-day configuration (Fig. 1D).

We also consider the case where Europa accreted both ice and hydrated silicates, which combines endmember B with endmember A and/or C. Given that the hydrated mineral assemblages in this study (~7 to 9 wt % H_2_O) already contain enough water to form the water layer, fluid contributions from ice melting and silicate dehydration may lead to an oversized ocean-ice shell, which poses another challenge. We discuss the prospect of other mechanisms to remove excess water in the “Processes not modeled” section.

Models of Europa’s thermal evolution are also sensitive to the timing of Europa’s formation relative to calcium-aluminum inclusions (CAIs), which were the first solids to have condensed in the solar system. Early in solar system history, sufficient amounts of ^26^Al were present to melt the silicates in even relatively small asteroids (*52*). However, because ^26^Al is very short-lived [half-life of 714 thousand years (ka)], the amount of heating falls rapidly in the first few millions of years. Canup and Ward (*49*, *53*) suggested that Europa (and the other Galilean satellites) formed shortly before Jupiter’s disc dissipated. From isotopic differences between meteorite groups, Kruijer *et al.* (*54*) note that Jupiter was still accreting substantial amounts of material until at least ~3 to 4 million years (Ma) after solar system formation. From this, we infer that Europa likely concluded its own accretion between ~3 and 5 Ma after CAIs.

**Fig. 2.**
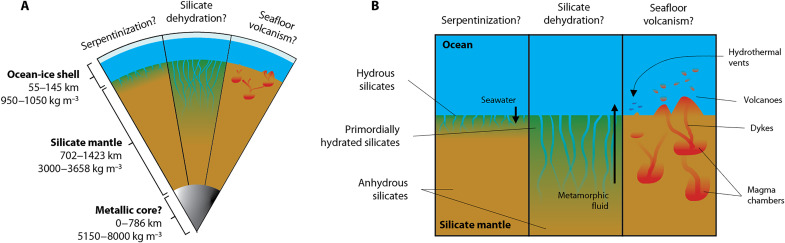
Observations support a partially or completely differentiated Europa, but available data have not revealed the ongoing geologic processes at the seafloor. (**A**) The NASA *Galileo* spacecraft detected an induced magnetic field at Europa, which is best explained by a global salty subsurface ocean (*5*, *6*). Evidence for a water shell, silicate mantle, and iron-rich core comes from models constrained by *Galileo* radio doppler data (*4*, *7*–*11*), although recent analyses admit the possibility that no metallic core exists (*43*). We estimate layer thicknesses for Europa using mass, radius, MoI, and assumed layer densities as constraints (see table S3). (**B**) Models of Europa’s interior structure permit a variety (but locally exclusive) set of geological processes. Fluid can aqueously alter ultramafic anhydrous rock at the seafloor through a process called serpentinization. However, Europa’s seafloor may never dehydrate if Europa accreted a hydrous mineral assemblage (Fig. 1B). Seafloor volcanism may occur if there is silicate melting at shallow depths and dike propagation between magma chambers and the ocean (*23*). Serpentinization, hydrothermal vent systems, and silicate volcanism may support ocean habitability by sustaining chemical disequilibria (*1*–*3*).

The thermal state of Europa’s rock-metal interior affects the seafloor conditions. Figure 2A shows our model of Europa’s interior structure based on the updated MoI value of 0.3547 ± 0.0024 (*43*). From existing gravity data, we do not know whether the silicates below the seafloor are heating up or cooling down today (Fig. 2B)—or whether those silicates are mostly hydrated or dehydrated. If the seafloor is cooling down, then serpentinization may hydrate the seafloor from above. Alternatively, if the seafloor is already hydrated but heating up, then fluid would mostly move upward into the ocean today. Pulses of heating in a dehydrated seafloor could also cause silicate melting and volcanism. Some recent studies favor seafloor volcanism under the assumption that Europa accreted silicate-metal differentiated (*22*, *45*). However, Europa’s seafloor may remain hydrated (i.e., chemically inactive) on geologic time scales, which may inhibit rock-water reactions that would otherwise produce heat and chemical energy for life (*55*). Ultimately, understanding when and where these processes occur is vital to modeling the habitability of the ocean, including how the prospects for life may change over geologic time.

In this study, we model the thermal evolution of Europa’s rock-metal interior assuming low accretional temperatures, as expected from the energetic arguments above. Our goal is to address three key questions: Could Europa experience enough silicate dehydration to form the ocean-ice shell? When did Europa start forming its metallic core (if it formed at all)? And what are the physical conditions at Europa’s seafloor today?

## RESULTS

### Thermal evolution modeling approach

We use one-dimensional thermal evolution models of Europa’s rock-metal interior to address three key aspects of the satellite’s internal history. First, we estimate the extent of silicate dehydration inside Europa. Second, we track when internal temperatures are high enough to melt Europa’s core-forming alloy and trigger metallic core formation. Third, we explore possible seafloor environments.

Our models feature four physical components: water-ice, hydrous silicates, anhydrous silicates, and metal. We define each component by its bulk thermophysical properties (see table S2), not by explicit chemical composition. Ice, rock, and free metal can coexist as a bulk mixture until high temperatures differentiate the components by melting one of the phases. As temperatures escalate with time, the thermophysical properties of the residual mixture changes as water leaves the underlying rock. Ice Ih melts at temperatures as low as ~250 K (*50*). Antifreezes (e.g., salts and ammonia) could lower the melting temperature of water-ice (see the “Processes not modeled” section). Our hydrated silicates contain 6.8 wt % H_2_O, which is consistent with Prinn-Fegley 2 (PF2) rock, a composition used to model icy bodies like Titan (*56*) and the Pluto-Charon system (*57*). Silicate dehydration occurs from ~550 to 900 K (*36*) and results in a denser anhydrous component (see table S2). We approximate metal as an Fe-FeS alloy whose densities and melting temperatures (~1250 to 1950 K) change with respect to sulfur content (*46*, *47*).

We model heat production and transport by solving the heat diffusion equation in spherical geometry, including thermal conduction, silicate dehydration, radiogenic heating, and a simplified model of tidal heating (see Materials and Methods). We choose a seafloor temperature of 250 K as the upper boundary condition for the rock-metal interior. In most of our models, we set the initial temperature of the interior to 250 K. We model radiogenic heating assuming chondritic abundances for short- and long-lived isotopes (table S1) (*58*). The abundance of the short-lived isotope (^26^Al) can decay by a few half-lives from its initial value in the solar system depending on Europa’s accretion time after CAIs, *t*_acc_ ~ 3 to 5 Ma.

We expect tidal dissipation to provide most of the energy supporting Europa’s current ocean, but many details are still unknown. The viscoelastic response time (i.e., Maxwell time) of water-ice is similar to Europa’s orbital frequency (*59*). Therefore, some studies assume that all or nearly all the tidal dissipation occurs in Europa’s ice shell (*59*, *60*). Given the large uncertainties in the interior rheology, however, substantial dissipation could occur in the rocky mantle (*61*). Coupled thermal-orbital evolution models suggest that Io and Europa may have experienced periods of oscillating orbital eccentricity and tidal heating rates, particularly in their early history (*59*, *62*). Here, we opt for a simplified treatment of tidal heating. We set a constant (with time and depth) tidal heating rate in the rock-metal interior, ${\dot{Q}}_{T}$. This approach allows us to assess the sensitivity of Europa’s internal evolution to different tidal heating scenarios.

A hydrated mineral assemblage constitutes some fraction of Europa’s bulk mass, which we denote as *X*. To calculate the thermophysical properties of our hydrated silicates, we assume that water in hydrated silicates is present in the mineral antigorite. For example, the aforementioned PF2 rock is 53.5 wt % antigorite (*56*, *57*). *X* = 1 if all silicates in our models are hydrated (i.e., saturated at 6.8 wt % H_2_O). In contrast, *X* = 0 implies that no water is found within the silicates, requiring that all of Europa’s water is present in the ocean-ice shell. We assume that all dehydrated fluid migrates to the ocean within one model time step, consistent with prior studies (*63*, *64*).

In addition to releasing water, silicate dehydration acts as a chemical heat sink. Dehydration of antigorite is a two-step process that first produces forsterite and talc and then enstatite. The total latent heat of dehydration adds up to 377 kJ kg^−1^ (*65*). We track the latent heat consumed that would otherwise warm the interior.

Overall, our models are most sensitive to four key parameters: Europa’s formation time relative to CAIs (*t*_acc_), the initial hydration state of accreted silicates (*X*), the abundance of sulfur in the Fe-FeS core-forming alloy (*f*), and the rate of tidal heating in the rock-metal interior (${\dot{Q}}_{T}$). Other uncertainties include Europa’s initial temperature (*T*_acc_), whether Europa accreted ice-rock differentiated or undifferentiated (Fig. 1, A and B), the exact thermophysical properties of the silicates, and the total mass of water in Europa. We test the effects of varying these parameters in our model—and find that they affect our conclusions less than the uncertainties on the four key parameters. Our Methods and Materials include detailed justifications of our assumptions and descriptions of all the governing equations.

### Example model

**Fig. 3.**
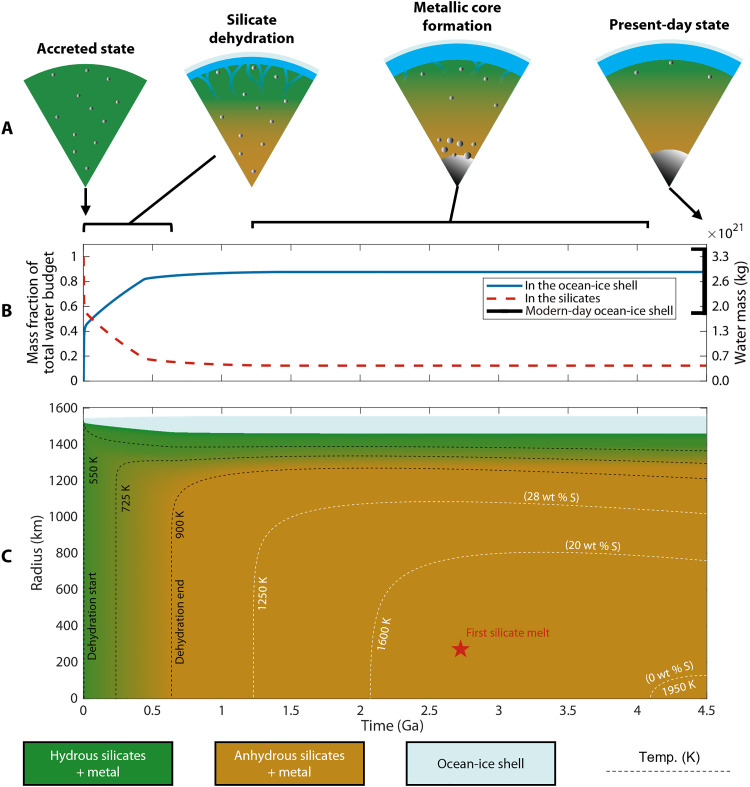
Europa released enough fluids from silicate dehydration to form the ocean-ice shell and experienced metallic core formation billions of years after accretion. The outermost silicates remain hydrated, demonstrating that radiogenic heating alone cannot lead to shallow silicate melting and volcanism if Europa’s silicates were primordially hydrated (Fig. 1B). (**A**) Cartoons of Europa’s internal structure and ongoing processes lined up with (B) and (C), both of which correspond to the nominal case. The horizontal brackets show the timing of ongoing silicate dehydration and when the temperatures are high enough to melt an Fe-FeS alloy, which may trigger metallic core formation. (**B**) The distribution of Europa’s total water budget over time suggests that most of Europa’s hydrocryosphere has a metamorphic origin. In this model, Europa is initially 6.8 wt % H_2_O which is consistent with “PF2” rock (*3*). Assuming that Europa’s MoI is 0.3547 ± 0.0024 (*31*), we self-consistently estimate the ocean-ice shell mass fraction to be 3.7 to 7.2 wt % H_2_O (see table S3) as shown by the vertical black bracket. (**C**) A plot of temperatures and hydration state, showing that Europa started forming its metallic core ~1.2 to 4.1 Ga after accretion. The horizontal and vertical axes represent time after accretion (Ga) and radial distance from Europa’s center (km), respectively. The solid colors represent the composition of a bulk material mixture. The dashed lines are temperature contours in kelvin. Black contour lines indicate temperatures where there is ongoing silicate dehydration, across 550 to 900 K (*1*). White contours represent temperatures where Fe-FeS alloys can melt, around 1250 to 1950 K (*7*, *13*). The red star marks the first silicate melt using the solidus for anhydrous peridotite (*66*). For this example, we use the following parameters: *t*_acc_ = 3 Ma after CAIs, *T*_acc_ = 250 K, *X* = 1, and ${\dot{Q}}_{T}$ = 0 W.

Figure 3 presents our “nominal case”—a model that captures all our main results, which we elaborate on in the following subsections. Figure 3A illustrates how Europa’s interior structure evolves during the model, as Fig. 1 foreshadowed. Figure 3B shows the calculated distribution of Europa’s water budget over time. The red and blue curves represent water in the rock-metal interior and ocean-ice shell, respectively, which add up to Europa’s total water budget. The black line brackets the mass of the present-day ocean-ice shell (~7% of Europa’s total mass), inferred from Europa’s mass, radius, and MoI of 0.3547 ± 0.0024 (*43*). Figure 3C shows how the temperature and composition of Europa’s interior evolve in tandem. The colors represent different mixtures of material components. The dashed lines are temperature contours in kelvin, drawn at the key thermal milestones in our models. Black contour lines represent the starting, middle, and ending temperatures of silicate dehydration. White contours represent Fe-FeS melting temperatures (i.e., start of metallic core formation) for alloys of 28 wt % S (eutectic), 10 wt % S, and 0 wt % S. We track the size of the (growing) ocean-ice shell but do not model its dynamics in detail. The red star shows where silicate melt can first occur according to the solidus for anhydrous peridotite (*66*). Figures S1 to S8 are the same as Fig. 3 (B and C) but with one parameter varied at a time with respect to the nominal case.

The nominal model starts at *t*_acc_ = 3 Ma with Europa’s interior assumed to be a mixture of hydrated silicates and metal (*X* = 1). We assume that no free ice exists in the interior, so all water is bound to hydrated rock. Silicate dehydration begins within a few million years due to rapid warming from the short-lived isotope ^26^Al. Almost all silicate dehydration takes place in the first ~0.5 billion years (Ga) of our model and discharges enough fluid to form Europa’s present-day ocean-ice shell. Meanwhile, the rock-metal interior shrinks as mass is transported to the ocean.

A thermal boundary layer grows from the seafloor downward into the rock-metal interior. Near the seafloor, conductive cooling leads to a near-linear rise in temperatures with depth. Closer to Europa’s center, temperatures are nearly constant with depth, rising with time as more heat is produced. In Fig. 3C, the growing boundary layer manifests as horizontal temperature contours that gradually extend deeper into Europa. It takes nearly all solar system history for thermal conduction to penetrate all the way to the center of the Europa.

Metallic core formation may start when temperatures are high enough to melt metal. The melting temperature, and therefore timing of metallic core formation, depends on the bulk sulfur content of the Fe-FeS alloy (*f*), which may in principle range from pure Fe (*f* = 0 wt % S) to the Fe-FeS eutectic (*f* = 28 wt % S). Previous internal models have used Fe–(10 wt %)S as an intermediate value supported by ordinary chondrite composition (*8*). The addition of sulfur lowers the melting point of pure iron from ~1950 K down to ~1250 K at the eutectic (*46*, *47*) at Europa’s central pressure (see the Supplementary Materials). If the metal has an Fe-FeS eutectic composition, then the core may start forming ~1.2 Ga after accretion. In contrast, a pure-Fe core would not start forming until ~4.1 Ga after accretion in the nominal model. In general, our models predict that metallic core formation begins billions of years after accretion.

Silicate melting first occurs deep within the interior—probably too deep to drive volcanic activity at the seafloor. Magma must migrate upward from the depth at which silicate melting occurs to form seafloor volcanoes. By modeling dike propagation, Bland and Elder (*23*) argued that Europa may experience cyclic volcanic episodes every several thousand years, but the conditions necessary for such volcanism are far from assured even if we assume a hot start [e.g., rock-metal differentiated after accretion, ~100 km lithosphere, and global melt production according to (*45*)] (*23*). If dikes form too frequently or numerously, then the magma flux into each dike will be too low to transport magma across a ~100-km lithosphere. Our nominal model, which assumes a cold start of 250 K, predicts that silicate melting starts at ~1200 km below the seafloor. Furthermore, less energy will be available for subsequent melting than hot start models.

Overall, we can divide the history of Europa’s thermal evolution and differentiation into a few stages. Before metallic core formation, the satellite slowly warmed up until the onset of silicate dehydration. The resulting metamorphic (supercritical) fluid interacted extensively with the surrounding host-rock before percolating to the ocean (*24*, *37*, *38*). These rock-water reactions operated at substantially higher pressure/temperature conditions than assumed in previous geochemical models that emphasize low temperature and pressure geochemistry (*13*, *24*–*27*). Metallic core formation also released gravitational potential energy as a heat pulse from dense metallic components migrated toward Europa’s center and rapidly warmed the deep interior. This slow evolution is in stark contrast with previous studies that assume that rock-metal differentiation began during or immediately after accretion (*44*, *45*, *62*, *67*).

### Metamorphic origin of the ocean

Slow internal heating may produce most or all of Europa’s ocean-ice shell mass through silicate dehydration if Europa accreted a hydrated mineral assemblage. Scientists have pondered a metamorphic origin for Europa’s ocean for at least 40 years because if Europa’s interior is composed of an anhydrous rock-metal mixture, the interior is large enough to consume the relatively thin ocean-ice shell (*24*–*27*, *37*, *38*, *51*, *68*). However, these studies acknowledged that the extent of silicate dehydration is unknown or assumed that Europa’s silicate interior completely devolatized due to metallic core formation. If Europa’s silicates retain a substantial amount of water, then the ocean-ice shell may not be entirely metamorphic in origin. We show that, assuming Europa accreted material with a similar bulk water content to its current water-rock ratio, the thermal evolution of Europa indeed causes enough silicate dehydration to form the present-day ocean-ice shell.

We find that a metamorphic origin for Europa’s ocean is plausible for a wide range of model parameters. The Supplementary Materials contain figures that illustrate our sensitivity tests. Figures S1 to S8 are analogous to Fig. 3 (B and C), but each supplementary model varies one parameter with respect to the nominal model. Increasing ${\dot{Q}}_{T}$ from 0 to 1 terawatt (TW) results in greater fluid production, but both models are consistent with Europa’s current ocean-ice shell mass (fig. S1). Decreasing *t*_acc_ from 3 to 5 Ma after CAIs results in lower internal heating and less silicate dehydration, but the low-heating model still produces enough fluid to account for Europa’s ocean-ice shell mass (fig. S2). Varying *T*_acc_ from 200 to 300 K results in the same amount of silicate dehydration as the outermost silicates remain cool and hydrated (fig. S4). Allowing Europa to accrete ice-rock undifferentiated negates the heating from short-lived radioactive isotopes, but results remain similar to the case with low radiogenic heating (fig. S5). Increasing the specific heat of anhydrous silicates from *c*_p_ = 900 to 1000 J kg^−1^ K^−1^ slows down the warming of the interior, but silicate dehydration still produces enough fluid to form the ocean-ice shell (fig. S6). Decreasing the thermal conductivity of anhydrous silicates from *k* = 4.2 to 3.0 W m^−1^ K^−1^ increases fluid production, but the total water yield still matches Europa’s ocean-ice shell mass (fig. S7).

Whether silicate dehydration can produce the correct amount of fluid to match Europa’s present-day ocean-ice shell depends on the water content of accreted silicates (figs. S3 and S8). We assume that no substantial volatile loss occurred after accretion due to the unavailability of sufficient energy (*69*). If Europa’s accreted silicates contain less water than Europa’s present-day water shell, then Europa must have accreted some water ice as well. If Europa accreted a mineral assemblage that is >9 wt % H_2_O (i.e., more hydrated than the rock used in all other models in this study), then our models predict that silicate dehydration may produce too much fluid to match Europa’s observed ocean-ice shell (fig. S8). Some geochemical studies explored Europa’s ocean composition considering a CI chondrite–like precursor, but they pointed out that their model water budgets exceed that of Europa’s current water shell (*24*, *38*). While Europa’s silicate mantle may retain some water, we agree that assuming a CI chondrite–like composition for Europa’s entire accreted material will result in oversized water shells given the extent of silicate dehydration in our models (>70 to 97 wt % of the rock-metal interior). However, we cannot rule out scenarios with some CI chondritic material accreted alongside other, less hydrated mineral assemblages.

### Timing of metallic core formation

Unlike large rocky planets, Europa could form its metallic core billions of years after accretion. The metallic core may form only after internal temperatures are high enough to melt Europa’s core-forming alloy and, if applicable, dehydrate the deep silicate interior. Here, we show that, if Europa accreted at low temperatures (e.g., ~200 to 300 K), then secular warming and silicate dehydration may delay metallic core formation on geologic time scales.

**Fig. 4.**
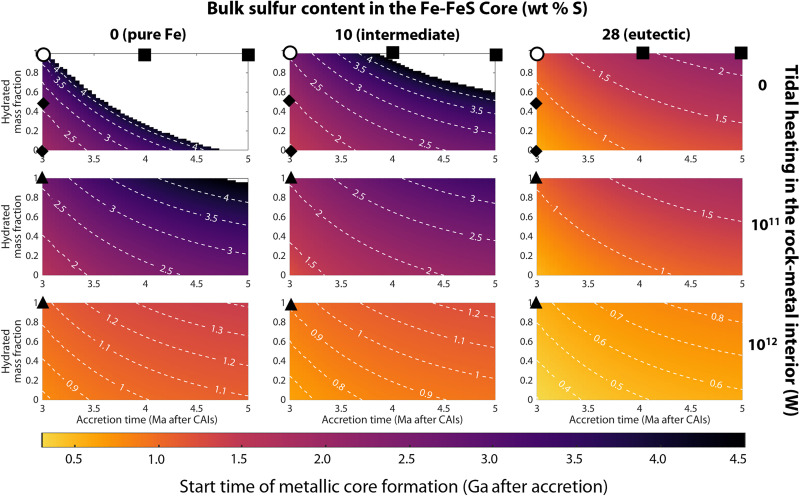
Europa’s metallic core formation likely initiates billions of years after accretion. Our parameter sensitivity analysis includes Europa’s accretion time *(t*_acc_), hydrated mass fraction (*X*), sulfur abundance in the Fe-FeS core (*f*), and total tidal heating in the rock-metal interior (${\dot{Q}}_{T}$) assuming that Europa’s initial temperature is *T*_acc_ = 250 K. The horizontal and vertical axes correspond to *t*_acc_ and *X*, respectively. The rows and columns represent variations in ${\dot{Q}}_{T}$ and *f*, respectively. The white circles represent the parameters of the example model in Fig. 3. The black triangles, squares, and diamonds represent parameters for the supplementary models in figs. S1 to S3, respectively. The timing of metallic core formation is most sensitive to uncertainty in *t*_acc_ and *X* (tied), *f*, and ${\dot{Q}}_{T}$ in increasing order. Our model only includes thermal conduction, although more efficient heat transport mechanisms such as silicate convection and heat-pipe volcanism would slow down the warming of the interior and further delay metallic core formation, thus strengthening our claim that metallic core formation happens late.

Our result of late metallic core formation holds for most of our explored parameter space. Figure 4 shows how the timing of metallic core formation depends on major model parameters: *t*_acc_, *X*, *f*, and ${\dot{Q}}_{T}$. The horizontal and vertical axes of each subplot are *t*_acc_ and *X*, respectively. The rows and columns represent ${\dot{Q}}_{T}$ and *f*, respectively. The colors and contour lines show the timing of metallic core formation in units of billion years (Ga). White space means that internal temperatures were never high enough to initiate metallic core formation. White circles all correspond to model parameters of the nominal case. The black triangles, squares, and diamonds represent model outputs of different parameter choices in figs. S1 to S3 (analogous to Fig. 3, B and C).

Figure 4 presents three choices for Europa’s Fe-FeS core composition (*f* = 0, 10, and 28 wt % S). Many studies have assumed the Fe-FeS eutectic for Europa’s core composition because this composition has the lowest melting point (*28*, *36*, *38*, *44*, *70*). However, the Fe-FeS eutectic contains an excess concentration of sulfur relative to chondrites. If one were to invoke chondritic material as an analog composition for Europa’s accreted material, then a value of *f* = 10 wt % S may be more plausible (*9*). Figure 4 shows that, if Europa’s Fe-FeS core contains less than 10 wt % S, then Europa may have spent most of its life without a fully formed metallic core, possibly to the extent that Europa is still differentiating today.

We further demonstrate late metallic core formation for other model parameter choices in the Supplementary Materials. The thermal evolution plots in figs. S1 to S8 are analogous to Fig. 3 (B and C), but each supplementary model varies one parameter with respect to the nominal model. Increasing tidal heating in our model from ${\dot{Q}}_{T}$ = 0 to 0.1 TW still results in metallic core formation times at least 1 Ga after accretion (fig. S1). Increasing Europa’s formation time from *t*_acc_ = 3 to 5 Ma will decrease internal heat production and further delay metallic core formation. Internal temperatures for the “low radiogenic heating case” (*t*_acc_ = 5 Ma) are never high enough to melt Fe-FeS alloys that are *f* < 17 wt % S (fig. S2). Increasing Europa’s accretional temperature from *T*_acc_ = 250 to 300 K will shift metallic core formation times by a few hundred million years, but metallic core formation starts at least a billion years after accretion nonetheless (fig. S4). Our ice-rock undifferentiated models result in similar thermal evolution and metallic core formation times as the low radiogenic heating case, so metallic core formation begins anywhere between 2.1 Ga and the present-day. Sulfur-poor cores may not form at all (fig. S5). Increasing the specific heat of anhydrous silicates from *c*_p_ = 900 to 1000 J kg^−1^ K^−1^ slows down the warming of the interior which further delays metallic core formation (fig. S6). Metallic core formation times do not perceptibly change in our models when varying the thermal conductivity of anhydrous silicates from *k* = 4.2 to 3.0 W m^−1^ K^−1^ (fig. S7). Increasing the bulk water content of Europa’s saturated hydrated silicates will increase the energy required to dehydrate the silicate interior, thus strengthening our argument for late metallic core formation (fig. S8).

Our models result in metallic core formation times that start <1 Ga after accretion only for a narrow set of model parameters. One scenario involves high tidal heating rates in the rock-metal interior. If we increase the tidal heating of our nominal model to ${\dot{Q}}_{T}$ = 1 TW, then our models suggest that metallic core formation may begin ~0.5 to 1 Ga after accretion (fig. S1C). Another scenario is to assume no silicate dehydration (*X* = 0) and high core sulfur content (*f* > 22 wt % S), which delay the formation of the metallic core by ~0.6 to 1 Ga in our models (fig. S3C). In both scenarios, metallic core formation is instead delayed by hundreds of millions of years. However, our models may underestimate the formation time of the metallic core because they do not include additional mechanisms of heat transport such as solid-state convection, which would further slow the warming of the interior. Ultimately, the vast majority of our models predict that metallic core formation starts billions of years after accretion.

### Seafloor conditions at present day

As discussed in Introduction, our models of Europa may predict one of the following processes today: serpentinization, silicate dehydration, and seafloor volcanism (Fig. 2). However, in most of our models, none of these processes are ongoing at the present day. If Europa accreted with hydrated silicates, then about 90% of all silicate dehydration takes place within the first ~0.5 to 1.5 Ga after the simulation start time. Afterward, the total dehydration rate gradually decreases over time. Our models do not generate any metamorphic fluid at present day, so silicate dehydration should be seen as an ancient process. Our common result is that the seafloor remains cool and hydrated at present day.

All our models disfavor seafloor volcanism if the silicate interior experiences ≤1 TW of tidal heating (Fig. 3 and figs. S1 to S8). We estimate when and where the silicates first start melting based on the solidus of anhydrous peridotite (*66*). Our models suggest that silicate melting, if it occurs at all, starts several hundreds to >1000 km below the seafloor. Such deep melting may require high magma fluxes and fracture toughness in the lithosphere for magma dikes to propagate to the seafloor (*23*). Silicate melting also acts as a heat sink, so our models may overpredict temperatures in Europa’s interior after melting occurs. We speculate that ongoing seafloor volcanism is unlikely, but more complex models that simultaneously implement tidal heating, silicate melting, magma transport, metallic core evolution, and other processes may refine our expectations (see the “Processes not modeled” section).

In the absence of seafloor volcanism, Vance *et al.* (*14*) explored whether thermal cracking may drive serpentinization at Europa. If the lithosphere is cooling, then the seafloor may become more brittle over time and accumulate residual stresses at grain boundaries, eventually leading to cracks that may expose dry rock for subsequent serpentinization. For cooling rates and grain sizes explored by Vance *et al.* (*71*), thermal fracture depths typically do not exceed ~50 km in Europa. However, if *X* = 1, then our models predict that the outer ~200 km of Europa’s silicate interior may never dehydrate (see Fig. 3 and figs. S1 to S8), implying that ~50-km fractures would expose rock that is already hydrated. Our models most favor serpentinization under certain conditions: Europa did not experience widespread aqueous alteration, experienced little warming from short-lived radioactive isotopes, and dissipates most of its tidal heating in the ice shell (e.g., *X* = 0, *t*_acc_ = 5 Ma, and ${\dot{Q}}_{T}$ = 0 to 0.1 TW). In any case, thermal fracturing may play a role in the seafloor’s evolution, especially if, for example, the effective grain size is relatively high or if warming can help generate deep fractures.

## DISCUSSION

### Ocean-ice shell chemistry and composition

Our work sets a geodynamic foundation for two key geochemical investigations of Europa’s ocean: (i) the timing of ocean formation and subsequent thermal evolution and (ii) the extent of silicate dehydration. In the first case, we show that silicate dehydration is an ancient process at Europa if it occurs at all. Early silicate dehydration would determine the initial composition of the ocean. The subsequent ocean composition would therefore evolve based on chemical fluxes across the ocean-ice and rock-ocean interfaces, the latter of which is unlikely for most of our models. In the second case, we show that up to 30% of the original rock mass may remain hydrated. The composition of a metamorphic ocean may vary if some volatiles are retained in the silicate mantle. Hydrated silicates are concentrated today at the cool and hydrated seafloor, thus limiting further water-rock reactions. Overall, our modeling provides a timeline for volatile release and implies limited rock-water reactions in the past few billion years.

Fluid released from silicate dehydration should experience changing physical conditions as it migrates to the seafloor. High temperature and pressure conditions of ~550 to 900 K and ~1 to 4 GPa imply that dehydrated fluid may be supercritical at the point of release. The temperature and pressure conditions will decrease as the (supercritical) fluid moves upward, potentially carrying aqueous solutes and redox-sensitive species along the way (*38*). At shallow depths, the fluid should be a cool liquid and encounter partially hydrated silicates. Rock-water chemistry should be limited at this stage given that the silicates will be partially or completely saturated with water. Once the deep interior fluid reaches the ocean, mixing between the released dehydrated fluid and existing ocean will foment chemical disequilibria—a compositional gradient that drives continuous chemical reactions. Our models predict that silicate dehydration should have finished a few billion years ago, so the fluid transport we describe here may not be ongoing. However, the absence of silicate dehydration does not prevent other processes, including other types of mass transfer, from contributing to ocean disequilibria.

Since the seafloor may remain hydrated throughout Europa’s lifetime, there may be limited (if any) serpentinization. One of our key results was that silicates may remain cool and hydrated a few hundred kilometers below the seafloor (see the “Seafloor conditions at present day”). The inaccessibility of anhydrous rock may prevent serpentinization which would otherwise supply heat and chemical energy to the deep ocean. Furthermore, a metamorphic ocean may result in a ~3- to 10-km layer of gypsum precipitate at the seafloor (*38*), which would further inhibit thermal cracking and aqueous alteration of olivine (*14*, *71*). Partial silicate dehydration, as predicted by many of our models, implies that the rock-metal interior does not continuously sustain chemical disequilibria. Therefore, a metamorphic ocean origin may instigate a “thermodynamics-driven extinction” unless the ice shell recycles ocean and surface material (*55*).

Our support for a metamorphic ocean origin may tie into the ongoing debate: Is Europa’s ocean dominated by Mg-sulfate or NaCl? There is disagreement on whether spectra of Europa’s surface provide evidence for the former (*29*–*32*) or latter (*39*–*41*) hypothesis. Geochemists and petrologists are also divided on this issue based on thermodynamic models (*24*–*27*, *38*, *68*, *72*). In the absence of ocean chemical data, arguments for specific ocean compositions must rely (for now) on assumptions on how elements migrate between the metallic core, silicate mantle, subsurface ocean, and ice shell over geologic time, which can be quite difficult. Our thermal models can guide future geochemical investigations on the timing and existence of different processes that influence ocean composition (*28*, *64*).

### Presence of a metallic core

While many studies have assumed Europa to have a metallic core (*4*, *8*–*11*, *28*, *44*, *45*, *61*, *67*, *70*), we show that metallic core formation is an expected but not guaranteed outcome (Fig. 4). Our models that never reach the high temperatures necessary to start metallic core formation require some combination of conditions: little to no tidal heating in the rock-metal interior, low sulfur abundance in the core-forming Fe-FeS alloy, and scarcity of short-lived radioactive isotopes. The existence of a metallic core would imply that those models likely underestimated the sources of energy in Europa’s interior. If Europa does have a metallic core, then we might also expect serpentinization via hydrothermal activity and, perhaps, seafloor volcanism.

There are at least two other reasons why Europa’s metallic core formation is important: (i) Metallic core formation extracts metal, sulfur, and other core-forming elements that would otherwise be susceptible to aqueous alteration, and (ii) metallic core formation introduces a late heat source that is rapid on geologic time scales. Regarding the latter, we estimate that metallic core formation is self-sustaining and may release enough heat to dehydrate the silicate interior, although the distribution of this core heat pulse requires additional modeling (see the Supplementary Materials).

### Implications for other icy moons

The underlying principle behind our models—low initial temperatures may beget substantial structural changes—may extend to other giant planet satellites. As argued in Introduction, icy moons have low mass relative to the terrestrial planets, so limited accretional heating should lead to cooler initial conditions than large rocky planets. What differs between each satellite is their individual history of tidal heating, radiogenic heating, and accreted composition. Consequently, the time scales between periods of elevated geologic activity (e.g., ocean formation) and quiescence vary across satellites, which is important for understanding the diversity in icy moon structures and habitable potential.

The Galilean moons exhibit a bulk density gradient that decreases with distance from Jupiter due to increases in ice-to-rock ratios (*7*, *73*). The innermost Galilean satellite is Io (~0 wt % water), followed by Europa (~5 to 9 wt % water), and then the ice-rich Ganymede and Callisto (~50 wt % water) (*4*, *7*–*9*, *43*, *73*). There is currently no consensus on whether the density differences among the Galilean satellites arose from their formation conditions (*49*, *74*–*79*) or subsequent evolutions (*24*, *38*, *80*) or both. Broadly speaking, our result that Europa’s ocean had a metamorphic ocean origin is consistent with many scenarios—and does not resolve this ongoing debate.

Our suggestion that silicate dehydration produced Europa’s entire ocean-ice shell raises another interesting question: Did Io ever have an ocean? There is no proven mechanism for removing a primordial water ocean at Io, but there are ideas. Some studies speculated that tidal heating dried out Io (*81*, *82*). However, there was likely not enough tidal dissipation energy to remove substantial amounts of water at Io (*69*). Long-term atmospheric loss (or sputtering) may remove volatiles (*83*), but sputtering was perhaps not intense enough to remove an entire ocean (*69*). More work is needed to understand the dehydration time scales at Io, what processes could remove this water inventory (including perhaps impacts and radiolytic loss), and if Io more plausibly formed from the anhydrous silicates that constitute most of its mass today.

Ganymede and Callisto require an exogenous ice source regardless of contributions from silicate dehydration. CI chondrites contain 11 to 18 wt % water, which is more than any other known meteorite class (*18*). Ganymede and Callisto contain even more water. Compatible with our assumption of low accretional heat, Mousis *et al.* (*79*) suggested that a phyllosilicate dehydration line in a gas-starved disc may explain the water-ice distribution among the Galilean satellites. In any case, metamorphic fluid still may contribute rock-derived solutes to their oceans, which affects ocean and ice shell composition.

A metamorphic ocean origin at Europa is potentially compatible with all Galilean satellites forming in the same circumjovian disc, given that disc temperature should decrease with distance from Jupiter. The inner Galilean satellites may have accreted hydrated silicates and metal, whereas the outer Galilean satellites also accreted volatile ices. If all Galilean moons accreted water-ice, then both Io and Europa require a mechanism to remove excess volatiles. The origin of the variable ice-to-rock ratio across the Galilean satellites remains an intriguing mystery.

## MATERIALS AND METHODS

### Model details

We opt for a one-dimensional treatment of Europa. The extent of silicate dehydration and timing of metallic core formation depend on Europa’s planet-wide evolution, for our results are not sensitive to local heating anomalies. In addition, the limited constraints we have on Europa—mass, radius, and MoI—are one-dimensional. A one-dimensional model thus accomplishes our scientific goals without the added complexity and runtime of a three-dimensional model.

We created a dehydration and thermal evolution model of Europa’s rock-metal interior by considering a time-dependent energy balance.

$$\dot{q}={\dot{q}}_{S}+{\dot{q}}_{R}+{\dot{q}}_{T}-{\dot{q}}_{L}$$ (1)

In units of power per volume, $\dot{q}$ is the net energy balance, $\dot{q}$_S_ is the secular heating term, $\dot{q}$_R_ is the radiogenic heating term, $\dot{q}$_T_ is the tidal heating term, and $\dot{q}$_L_ is the dehydration term. Silicate dehydration consumes latent heat, thus $\dot{q}$_L_ acts as an energy sink. Expanding each term and dividing by ρ*c*_p_ results in the heat diffusion equation.

$$\dot{T}=\frac{1}{\rho c_{p}r^{2}}\frac{\partial }{\partial r}\left({kr}^{2}\frac{\partial T}{\partial r}\right)+{\dot{T}}_{R}+{\dot{T}}_{T}-{\dot{T}}_{L}$$ (2)

Here, $\dot{T}$ is the net change in temperature over time (K s^−1^), ρ is the rock density (kg m^−3^), *c*_p_ is the specific heat capacity (J kg^−1^ K^−1^), *r* is the radial distance from Europa’s center (m), *k* is the thermal conductivity (W m^−1^ K^−1^), *T* is the temperature (K), *Ṫ*_R_ is the temperature increase due to radiogenic heating (K), *Ṫ*_T_ is the temperature increase due to tidal heating (K), and *Ṫ*_L_ is the warming mitigated by silicate dehydration (K). The initial and (seafloor) boundary conditions were set to 250 K. We did not track the thickness of the subsurface ocean or compute temperature profiles in the ice shell, since they do not affect the evolution of the rock-metal interior given our constant-temperature boundary condition. Our approach was to model only the factors that may substantially affect our key questions stated in Introduction (see the “Processes not modeled” section).

We calculated *Ṫ*_R_ using the following equation

$${\dot{T}}_{R}=\sum_{n}\frac{H_{0,k}}{c_{p}}e^{-\lambda_{k}t}$$ (3)

Here, *H*_0_ is the specific heat production (W kg^−1^), λ is a decay constant (s^−1^), and the *n* subscript indexes through the radioactive isotopes included in table S1. Radiogenic heating includes contributions from long-lived radioactive isotopes (^235^U, ^238^U, ^232^Th, and ^40^K) (*84*) and short-lived radioactive isotopes (^26^Al, ^60^Fe, and ^53^Mn) (*63*, *85*, *86*). ^26^Al has a half-life of 7.16 × 10^5^ years (*63*), so ^26^Al abundance is sensitive to our uncertainty in Europa’s accretion time. We let *t*_acc_ = ~3 to 5 Ma after CAIs to span nearly three half-lives of ^26^Al, the most powerful isotope. Therefore, variations in *Ṫ*_R_ are primarily due to ^26^Al abundance with respect to *t*_acc_.

We calculated *Ṫ*_T_ using the following equation

$${\dot{T}}_{T}=\frac{{\dot{Q}}_{T}}{Mc_{p}}$$ (4)

Here, ${\dot{Q}}_{T}$ is the total tidal dissipation in the silicate interior (W), and *M* is the Europa’s total mass (kg). We treated ${\dot{Q}}_{T}$ as a constant throughout time, ranging from 0 to 1 TW. Note that ${\dot{Q}}_{T}$ is constant, whereas *M* decreases by ~0 to 5 wt % depending on the extent of silicate dehydration. Tidal dissipation in the silicate interior therefore increases with silicate dehydration in our models, but this effect is minor given that our uncertainty in tidal heating may vary by 12 orders of magnitude. See the “Processes not modeled” section for consequences of simplified tidal heating.

We calculated *Ṫ*_L_ using the following equation

$${\dot{T}}_{L}=-L\frac{dX}{dt}$$ (5)

Here, *L* is the latent heat of dehydration (J kg^−1^). Note that *dX/dt* is a nonpositive value, since silicate dehydration reduces *X*. We assumed that silicate dehydration was a protracted process that occurs at 550 to 900 K (*36*). We further assumed that silicate dehydration proceeds linearly across this temperature range, meaning that the mass of fluid released per kelvin of heating is constant. The thermophysical properties of silicates change with the degree of dehydration (table S2). Silicate dehydration should release fluid mass and leave behind denser, anhydrous rock such that the overall size of Europa increases while the rock-metal interior contracts. See the “Numerical implementation” section for a detailed treatment of Eq. 5.

Metallic core formation begins once temperatures are warm enough to melt the core-forming metal alloy. Assuming hydrostatic equilibrium, we found that Europa’s central pressure before rock-metal differentiation is ~3.8 GPa. The central pressure should increase to ~4 to 6 GPa depending on the degree of dehydration and the size of Europa’s metallic core. For simplicity, we considered the liquidus of an Fe-FeS alloy for 4 GPa (*46*). An Fe-FeS core with 0, 10, and 28 wt % S starts melting around 1950, 1750, and 1250 K, respectively. Once internal temperatures reached the relevant liquidus, we assumed that metallic core formation begins.

### Numerical implementation

We used a finite-element adaptive grid and the discretized heat diffusion equation as derived in (*87*) but with radiogenic heating, tidal heating, and silicate dehydration. The density (ρ), specific heat (*c*_p_), grid cell thickness (Δ*z*), and thermal conductivity (*k*) change based on the extent of hydration (*X*) in the grid cell. Equation 6 gives the temperature change at each grid cell due to thermal conduction and internal heating.

$$\text{Δ}T_{j}=\text{Δ}t\left[\frac{-2}{\rho_{j}c_{p_{j}}\text{Δ}z_{j}r_{j}^{2}}\left(r_{j+\frac{1}{2}}^{2}\frac{T_{j+1}-T_{j}}{\frac{\text{Δ}z_{j+1}}{k_{j+1}}+\frac{\text{Δ}z_{j}}{k_{j}}}-r_{j-\frac{1}{2}}^{2}\frac{T_{j}-T_{j-1}}{\frac{\text{Δ}z_{j}}{k_{j}}+\frac{\text{Δ}z_{j-1}}{k_{j-1}}}\right)+{\dot{T}}_{R}+{\dot{T}}_{T}\right]$$ (6)

The subscript *j* is the grid cell index, and Δ*t* is the model time step set by the Courant condition, $\text{Δ}t\leq \frac{{\left(\text{Δ}z\right)}^{2}}{3K}$, where $K=\frac{k_{c}}{\rho c_{p}}$ is the thermal diffusivity (m^2^ s^−1^). For readability in the following equations, assume the current grid cell and time step—*i* and *j*, respectively—unless otherwise specified. The radiogenic and tidal heating at each grid cell can be computed using Eqs. 3 and 4. However, silicate dehydration may suppress the warming predicted in Eq. 6 (see below).

Equation 7 gives the energy change at grid cell *j* due to thermal conduction and internal heating.

$$\text{Δ}q={mc}_{p}(T_{i+1}-T_{i})$$ (7)

If silicate dehydration is ongoing (i.e., *T =* 550 to 900 K), then we must further partition the thermal energy Δ*q* at grid cell *j* into a secular and latent term.

$$\text{Δ}q=m_{i}{\bar{c}}_{p}(T_{i+1,}-T_{i})+dm_{h}L$$ (8)

$$f_{S}\text{Δ}q+f_{L}\text{Δ}q$$ (9)

Here, *dm_h_* is the mass of hydrated silicates undergoing devolatization (kg), and *L* is the latent heat of dehydration (J kg^−1^). Here, *f*_S_ and *f*_L_ are coefficients that describe the ratio of secular warming to latent heating. The average specific heat capacity, thermal conductivity, and density are based on the proportion of hydrous to anhydrous silicates (table S2). We assumed that silicate dehydration progresses linearly over the temperature range that hydrated silicates are expected to destabilize. We assume *L =* 202 kJ kg^−1^ which is consistent with PF2 rock (table S2). We solve for the secular warming and latent heat terms in Eq. 9 by obtaining coefficients *f*_S_ and *f*_L_, respectively, using a bisection root-finding algorithm.

Pairing Eq. 8 and Eq. 9, one can solve for *dm_h_* and *T* at grid cell *j* for each time step.

$${dm}_{h}=\frac{f_{L}\text{Δ}q}{L}$$ (10)

$$T_{i+1}=\frac{f_{S}\text{Δ}q_{i}}{m_{i}\bar{c_{p,i}}}+T_{i}$$ (11)

At any given moment, the mass of hydrated silicates (*m_h_*) within a parcel is *m_h_ = Xm* where *X* is the hydrated mass fraction in the silicates. We then computed hydrated mass fraction of the rock parcel at grid cell *j*.

$$X_{i+1}=\frac{X_{i}m_{i}-dm_{h,i}}{m_{i}-wdm_{h,i}}$$ (12)

*w* is the water content of our hydrous silicate endmember expressed as a mass fraction. Equation 12 implies that the ocean-ice shell gains *wdm_h_* of fluid in each time step. We assume that the wt % of H_2_O in the silicates scales linearly with *X* from 0 to 1. We solve for *f*_S_ and *f*_L_ in Eq. 9 at the start of each thermal simulation, whereas we solve Eqs. 10 to 12 every time step when silicate dehydration occurs.

The size of the rock-metal interior shrinks with silicate dehydration to conserve mass. Returning to grid notation with index *j* and dropping the *i* subscripts for readability, the radial position of each grid at *i* + 1 can be computed as

$$r_{j+1}={\left[r_{j}^{3}+\frac{3}{4\pi }\left(\frac{X_{j+1}m_{j+1}}{\rho_{h}}+\frac{(1-X_{j+1})m_{j+1}}{\rho_{d}}\right)\right]}^{\frac{1}{3}}$$ (13)

The updated density of each grid cell can be easily computed given that mass is conserved and Eq. 13 updates the volume of each cell.

### Processes not modeled

The purpose of this subsection is to discuss processes that may be relevant but were not included in our model. Our approach is to implement only the processes and variables necessary to address our key questions: Could Europa form its ocean-ice shell through silicate dehydration? When did Europa’s metallic core start forming? What are the possible seafloor conditions today? In general, our results are robust to the model assumptions that we make.

Our models focus on the thermal evolution of the rock-metal interior, which is independent of ocean thickness. We assume a constant boundary condition of 250 K at the seafloor, effectively assuming an ocean is always present. The thickness of the ocean does not affect our models’ seafloor boundary condition, so our results remain unchanged.

We assume that the ocean-ice shell composition is dominated by water, such that its melting point is that of water-ice. Our main model results do not change when decreasing our (seafloor) boundary condition, which could represent the effects of antifreezes such as salts and ammonia. Lower temperature boundary conditions should lead to predictions of a colder Europa, thus strengthening our claim that Europa’s rocky interior may be cool, partially hydrated, and late to differentiate. Even if our initial and boundary condition is 176 K (i.e., the melting temperature of ammonia-rich ice), silicate dehydration may still produce an ocean.

We simplified tidal heating to be a constant value with time and depth. Europa currently experiences a mean motion resonance with Io and Ganymede, which enhances tidal heating over geologic time. The thermal-orbital evolution of Galilean satellites can be quite complex. Europa may have undergone periods of oscillatory and near-equilibrium tidal heating (*45*, *62*). Such cyclic tidal variations may induce pulses of warming and, if applicable, silicate volcanism (*45*). However, our results are not sensitive to short-term variations in tidal heating because the timing of silicate dehydration and metallic core formation depends on cumulative heating. Our models do not include depth-dependent tidal heating. Hydrated silicates likely have lower viscosity than anhydrous silicates, meaning that tidal heating could be deposited preferentially in the hydrated zone beneath the seafloor. If so, then the chances of modern serpentinization are higher than our models predicted.

Solid-state convection would suppress the warming of the interior and further delay differentiation, which strengthens our argument for late metallic core formation. Convection transports heat more effectively than thermal conduction. However, modeling convection requires additional poorly known parameters such as viscosity. Our work shows that Europa’s cold start may delay metallic core formation by billions of years without the aid of silicate convection.

The metallic core formation process itself is not included in our modeling, but we can estimate its effect on the present-day conditions at the seafloor. Metallic core formation may act as a heat source because the sinking of dense metal releases gravitational potential energy. While the total energy released from metallic core formation is small relative to Europa’s total radiogenic and tidal heating, Europa’s core forming heat pulse may be rapid on geologic time scales (see the Supplementary Materials). Still, the part of Europa that participates in metallic core formation is probably deep in the rocky interior, so Europa’s core-forming heat pulse may have a limited effect on the seafloor environment.

Our support for a metamorphic ocean origin and late metallic core formation remains the same if we were to implement silicate melting. Unlike metallic core formation, silicate melting acts as an energy sink and may suppress the subsequent warming of the Europan interior. If ~40% of the silicate interior melts, then solid metal may sink downward to form a metallic core (*88*) as opposed to liquid metal flowing to Europa’s center. However, silicate melting and the formation of an S-deficient metallic core occur well after Europa’s internal temperatures reach the Fe-FeS eutectic which we show to be at least 1 Ga after accretion in many models (Fig. 4). We chose not to model silicate melting in detail because doing so would make our models more complex without changing our result that metallic core formation may be delayed by >1 Ga.

We assume that no volatile loss occurred after accretion. Modern observations are compatible with an excess amount of metamorphic fluid if volatiles escaped to space. Such volatile loss is beyond the scope of our work but highlights an interesting discussion on whether Io had an ancient water ocean (see the “Implications for other icy moons” section).

## Supplementary Materials

### This PDF file includes:

Supplementary Text Figs. S1 to S10 Tables S1 to S3 References
