# Supplementary material for: Slow evolution of Europa’s interior: metamorphic ocean origin, delayed metallic core formation, and limited seafloor volcanism
Source: Sci Adv. 2023 Jun 16;9(24):eadf3955. doi: 10.1126/sciadv.adf3955 (PMC10275592; doi:10.1126/sciadv.adf3955)
Supplement: Supplementary file 1 — Supplementary Text Figs. S1 to S10 Tables S1 to S3 References [file sciadv.adf3955_sm.pdf]

Science Advances
AAAS

Supplementary Materials for

Slow evolution of Europa's interior: metamorphic ocean origin, delayed metallic core formation, and limited seafloor volcanism

Kevin T. Trinh *et al.*

Corresponding author: Kevin T. Trinh, kttrinh1@asu.edu

Sci. Adv. **9**, eadf3955 (2023)
DOI: 10.1126/sciadv.adf3955

This PDF file includes:

Supplementary Text
Figs. S1 to S10
Tables S1 to S3
References

Supplementary Text

Additional model details and parameter sensitivity

We presented calculations using a dehydration and thermal conduction code in the main text. Here, we present a table of short- and long-lived isotopes used (Table S1), thermal parameters of our nominal model (Table S2), and comparisons of the nominal model (Figure 3) to differences in tidal heating in the rock-metal interior ($\dot{Q}_T$) (Figure S1), accretion time (t_{acc}) (Figure S2), initial hydration (X) (Figure S3), accreted temperature (T_{acc}) (Figure S4), accreted differentiation state (Figure S5), specific heat of anhydrous silicates (c_{pd}) (Figure S6), thermal conductivity of anhydrous silicates (k_{dehy}) (Figure S7), and water mass fraction of hydrated silicates (w) (Figure S8). We also describe the interior structure modeling in Figure 1, estimate water-ice phase stability of accreted Europa (Figure S9), plot the Fe-FeS liquidus as a function of sulfur content, depth, and pressure (Figure S10), and an estimate on energy released from metallic core formation.

Interior structure

Here we describe the modern interior structure model shown in Figure 1. Models of Europa's interior structure require that we know at least three things: mass, radius, and the normalized moment of inertia (MoI)—a single scalar value that non-uniquely describes the mass distribution

inside a body. The NASA *Galileo* spacecraft encountered Europa 11 times from December 1996 to September 2003, up to six of which were used to model the moon's mass and moment of inertia (*4*, *43*).

Without any *a priori* knowledge of the moon's interior structure, one can start simple and model Europa as a two-layer body: a rock-metal interior and ocean-ice shell. However, two-layer models have resulted in rock-metal interior densities that are higher than the bulk density of the neighboring anhydrous moon, Io (*4*).

A more complex but plausible scenario involves Europa segregating its rock-metal interior into a metallic core and silicate mantle. Three-layer models of Europa cannot provide unique solutions to layer thicknesses and densities. Instead, one may calculate the range of plausible layer sizes with assumed densities or vice versa. Many three-layer models of Europa assume hydrostatic equilibrium and thus are constrained by an MoI of 0.346 ± 0.005 (*4*, *7*, *10*, *11*). However, a recent reappraisal of *Galileo* radio doppler data without the assumption of hydrostatic equilibrium estimated Europa's MoI to be 0.3547 ± 0.0024 (*43*). This suggests a thinner ocean-ice shell and less dense rock-metal interior.

Here we model Europa as a three-layer body using the updated MoI and compare our results with previous studies in Table S3. As predicted by (*43*), our estimates for Europa's metallic core radius and ocean-ice shell thicknesses are smaller than that of previous studies assuming hydrostatic equilibrium. Additionally, we attribute our smaller layer sizes to our assumed density ranges: $\rho_{ocean\text{-}ice}$ = 950–1050 kg m^{-3}, ρ_{mantle} = 3000–3660 kg m^{-3}, and ρ_{core} = 5150–8000 kg m^{-3}. The ocean-ice shell and metallic core density ranges match that of (*4*). However, our minimum density for the silicate mantle is consistent with a rock-metal differentiated Europa. The upper bound of our density range exceeds that of bulk Io, which is thought to be anhydrous. However, metallic core formation should extract dense components from the mantle, so our upper bound on ρ_{mantle} is conservative.

Energy to warm/melt primordial water-ice

Here we elaborate on the endmember scenario where Europa starts out ice-rock undifferentiated (Figure 1A), which ultimately does not change our results. The energy required to raise the interior from Europa's initial temperature to the melting temperature of water-ice determines the delay time (i.e., the start time in our models). If Europa formed as a bulk mixture of ice, rock, and metal, then some of Europa's primordial ice may exist as a high-pressure polymorph, which can have elevated melting temperatures.

Temperature and pressure conditions throughout Europa's interior allow for liquid water and ice Ih–XIII to be stable (Figure S9). A previous study (*50*) provides a phase diagram of liquid water and ice Ih, II, III, V, VI, VII, VIII, and X based on a mathematical treatment of experimental data at 0–2500 K and 0.01–100 GPa which span conditions present in icy satellites. While uncertainty in Europa's initial T-P conditions allow for a wide variety of water-ice phases to be simultaneously stable, ice VI is typically the most abundant water-ice phase and comprises ~50% of Europa's primordial ice mass.

If Europa's primordial water-ice makes up ~10% of the satellite's total mass, the energy required to warm up primordial ice to liquid water phase boundary is roughly 5×10^5 J kg^{-1}, 2.5 $\times 10^5$ J kg^{-1}, and 7×10^3 J kg^{-1} for initial temperatures of 200 K, 250 K, and 350 K, respectively. The latent heat of melting for Ih to liquid water is 344 kJ kg^{-1}. We do not have the latent heat of melting for high-pressure ice phases transitioning into liquid water. If we further assume that all of Europa's primordial ice will melt with a latent heat of melting that is like that of ice Ih, then

only ~3 × 10^4 J kg^{-1} is needed to melt such ice which is an order of magnitude less than the latent heat of antigorite dehydration of 377 kJ kg^{-1} (*91*). However, the heat needed to warm up primordial water-ice to the liquid water phase transition temperature is comparable to the energy needed to devolatize Europa's hydrated silicates. Therefore, we ignore the heat required to melt primordial ice but consider the energy required to warm such ice to the liquid phase transition temperature. We explore an undifferentiated ice-rock-metal state for Europa's accreted state and subsequent thermal evolution.

Fe-FeS liquidus

The composition of Europa's Fe-rich core dictates the size of the metallic core and the temperatures required to initiate rock-metal differentiation. Since the exact composition of Europa's metallic core is unknown, we approximate the metallic core composition to be an Fe-FeS alloy which is often used in gravity-constrained models of Europa's interior structure (see *Interior structure modeling)*. See Figure S10B for the Fe-FeS liquidus temperatures as a function of temperature, pressure, and radial depth.

We refer to (*46*) for our interpolation of the Fe-FeS liquidus temperatures at < 10 GPa, which is minimum at the eutectic (i.e., ~28 wt.% S at Europa's central pressure of 3.8 GPa prior to rock-metal differentiation). On the Fe-rich side of the eutectic, an Fe-FeS eutectic core results in the largest core size and the lowest liquidus temperatures (~1263 ± 25 K) (*46*). On the other hand, the pure Fe liquidus is about 1940 K and results in a small metallic core that forms late. Figure S10a shows the Fe-rich side of the Fe-FeS liquidus at 3.8 GPa using Equation 29 from (*46*).

Energy from metallic core formation

The energy released from metallic core formation, ΔE_G, is the difference in gravitational binding energy between its undifferentiated and differentiated states. Gravitational binding energy can be represented as

$$E_G = G\int \frac{mdm}{r(m)} \quad (7)$$

where E_G is gravitational binding energy (J), G is the gravitational constant (m^3 kg^{-1} s^{-2}), m is mass enclosed (kg), and r is radial distance (m) as a function of mass. Therefore, the gravitational binding energy of a homogenous sphere is

$$E_{G,undiff} = \frac{-3GM^2}{5R} \quad (8)$$

The gravitational energy of a differentiated two-layer sphere is the sum of internal energies due to a sphere and shell (i.e., metallic core and silicate mantle). Here we provide an analytical derivation that we verified numerically.

Given that we already have the binding energy for a sphere, we can redefine our integral limits to solve for the energy of a shell.

$$E_{G,shell} = -\int_{m_c}^{M} \frac{Gmdm}{r(m)} \quad (8)$$

$$= -G \int_{m_c}^{M} \frac{mdm}{\left(r_c^3 + \frac{3m}{4\pi\rho}\right)^{\frac{1}{3}}}$$

Let $a = r_c^3$ and $b = \frac{3}{4\pi\rho}$.

$$E_{G,shell} = -G \int_{m_c}^{M} \frac{mdm}{(a + bm)^{\frac{1}{3}}} \quad (9)$$

Now substitute using $u = m$ and $dv = (a + bm)^{-1/3}\, dm$.

$$E_{G,shell} = -G \left(\frac{3m}{2b}(a + bm)^{\frac{2}{3}} - \int \frac{3}{2b}(a + bm)^{\frac{2}{3}} dm \right) \quad (9)$$

$$= -G \left(\frac{3m}{2b}(a + bm)^{2/3} - \frac{9}{10b^2}(a + bm)^{\frac{5}{3}} \right)$$

Combining the energy of the differentiated metallic core and silicate mantle gives us

$$E_{G,diff} = \frac{-3GM^2}{5R} - G \left[\frac{3m}{2b}(a + bm)^{\frac{2}{3}} - \frac{9}{10b^2}(a + bm)^{\frac{5}{3}} \right]_{m_c}^{M} \quad (9)$$

where M is mass of the rock-metal interior; R is the radius of the rock-metal interior (m); r_c is the radius of the metallic core, and ρ_c and ρ_m are the metallic core and silicate mantle densities, respectively. We find that ΔE_G is maximized for the largest expected metallic core of ~50% total radius and 5150 kg m^{-3} density (*4*), yielding ~ 3.9×10^{27} J. Metallic core formation could be a self-sustaining process if the core-forming energy is deposited into the metal, providing up at least double the energy per mass than the latent heat of fusion for pure iron (*92*).

The heat from metallic core formation may further dehydrate the silicate mantle using Eq. 4. Importantly, f_S and f_L remain constant assuming the thermophysical properties of our hydrous/anhydrous mineral assemblages and the temperature range of silicate dehydration does not change. This process may release a pulse of fluid

$$m_{pulse} = \frac{f_L \delta E}{L} \quad (10)$$

given that m_{pulse} does not exceed the mass of remaining hydrated silicates. However, the distribution of core-forming heat is beyond the scope of this study.

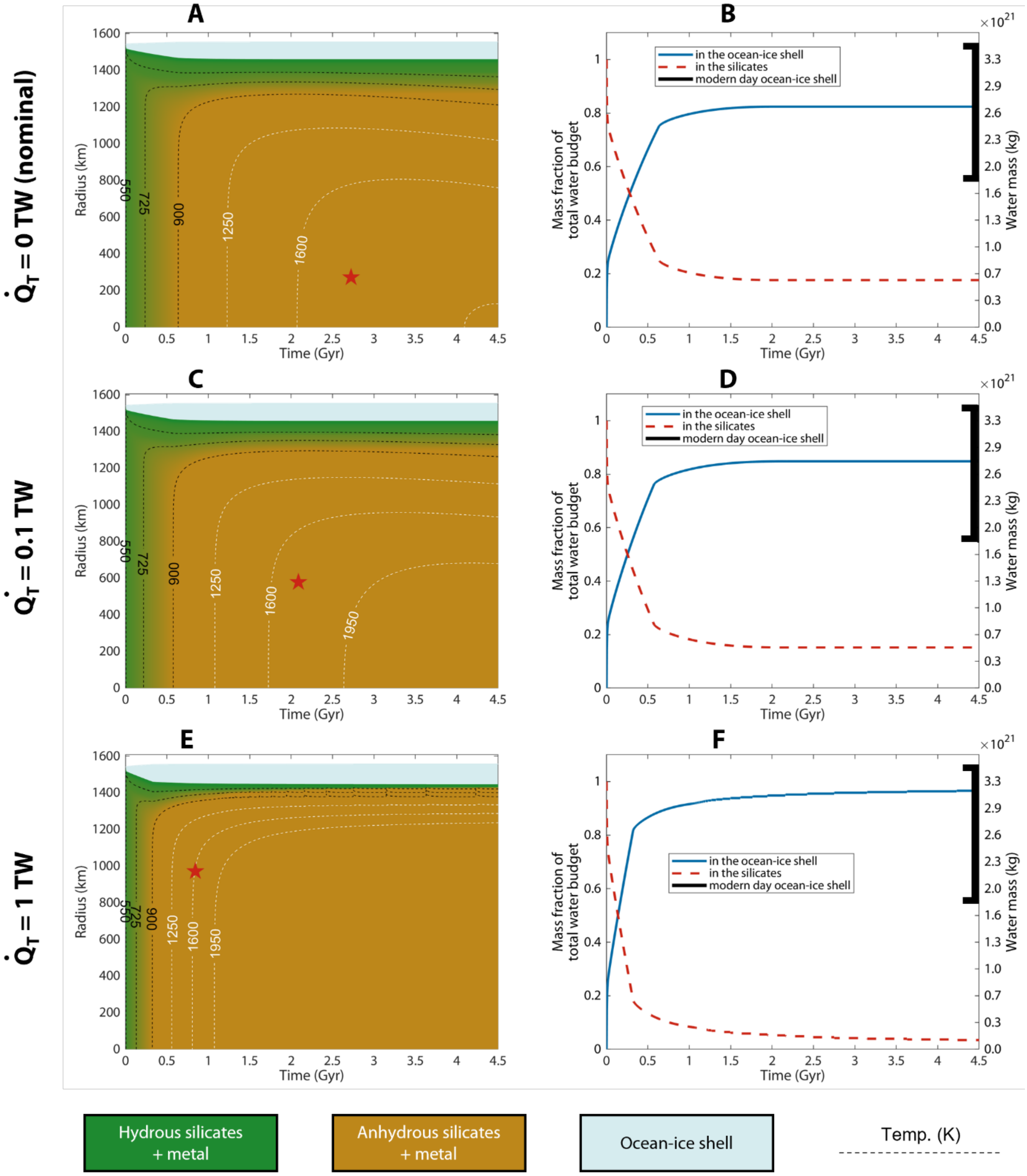

Fig. S1. Model results are most sensitive to tidal heating in the rock-metal interior ($\dot{Q}_T$) compared to all other parameters discussed in our work, but metallic core formation still may start billions of years after accretion. Europa releases enough fluid from silicate dehydration to form the ocean-ice shell. Silicate melting still occurs several hundred kilometers below the seafloor with $\dot{Q}_T$ = 1 TW, which may hinder volcanism. From the top to bottom row, the models vary only by tidal heating in the rocky interior: $\dot{Q}_T$ = 0 TW (nominal) (**A**–**B**), 0.1 TW (**C**–**D**), and 1 TW (**E**–**F**). The nominal model includes no tidal heating in the rock-metal interior, thus implying that all tidal heat is dissipated in the ice shell. The distribution and magnitude of

tidal heating in Europa is unknown. One could scale down tidal dissipative models for Io to Europa if both bodies have similar Im(k) values which results in $\dot{Q}_T = 5.5$ TW, though Europa is probably less dissipative than Io (*60*). Additionally, our model starts to break down when radiogenic and tidal heating are simultaneously high ($t_{acc} < 3$ Myr and $\dot{Q}_T = 1$ TW). This suggests that a purely conductive model cannot describe heat transport at Europa for such high heating cases. However, convection, while not implemented in our model, would suppress the warming of the rock-metal interior and further delay metallic core formation, thus strengthening our argument that metallic core formation happens late. All figure labels and symbols are the same as Figure 3 in the main text. Temperature contours past 1950 K are not shown.

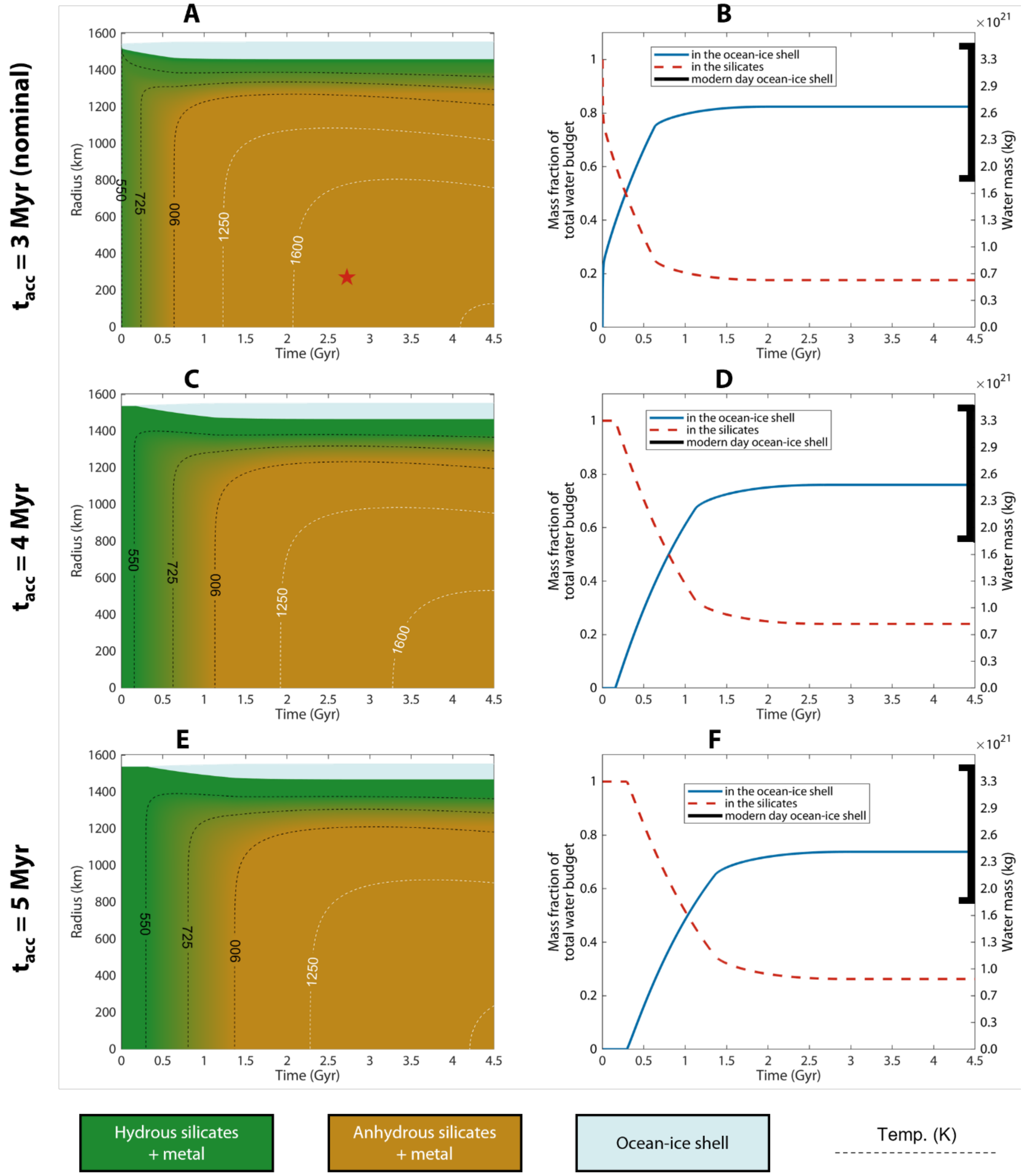

Fig. S2. Increasing Europa's accretion time (t_{acc}) from the nominal model strengthens our results. Europa releases enough fluid to form the ocean-ice shell, metallic core formation starts billions of years after accretion, and the outermost silicates remain hydrated. Silicate melting does not occur unless Europa forms early. From the top to bottom row, the three models presented vary only by Europa's accretion time: t_{acc} = 3 Myr (nominal) (**A**–**B**), 4 Myr (**C**–**D**), and 5 Myr (**E**–**F**). We explored this time range because Jupiter formed < 0.9 Myr after calcium-aluminum inclusions (CAIs) and maintained a gas-starved disc for 3–4 Myr (*49*). All figure

labels and symbols are the same as Figure 3 in the main text, though the Fe-FeS liquidus sulfur content are now summarized in Figure S10.

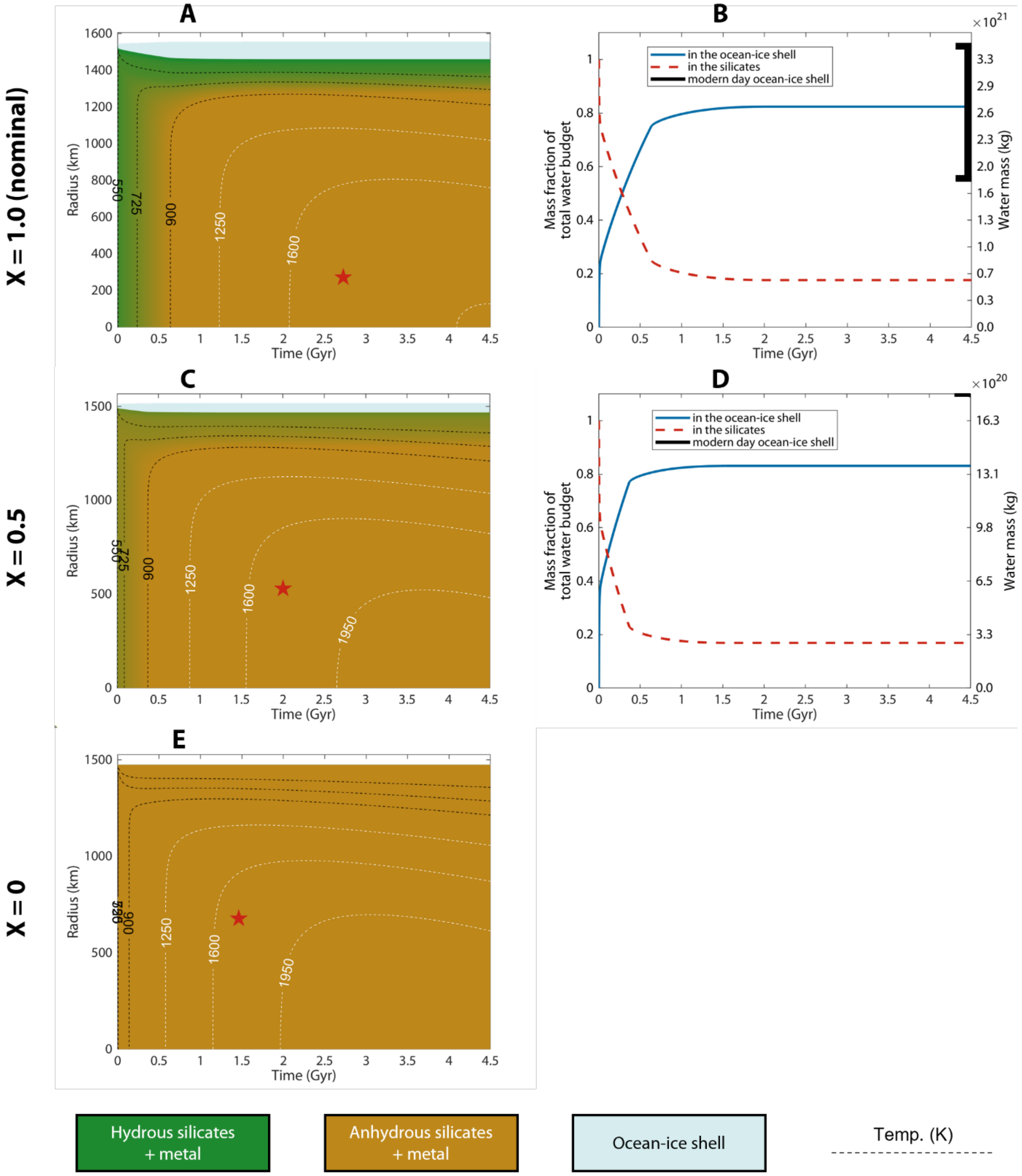

Fig. S3. Decreasing the initial abundance of hydrated silicates *(X)* facilitates the warming of the interior, but metallic core formation tends to start billions of years after accretion. Without tidal heating, our models suggest that metallic core formation begins no earlier than half a billion years. Silicate melting occurs several hundreds to ~1300 km from the seafloor. From the top to bottom row, the models presented vary only by hydrated mass fraction of accreted silicates: X = 100% (nominal) (**A**–**B**), 50% (**C**–**D**), and 0% (**E**). All figure labels and symbols are the same as Figure 3 in the main text, though the Fe-FeS liquidus sulfur content are now summarized in Figure S10. Temperature contours past 1950 K are not shown.

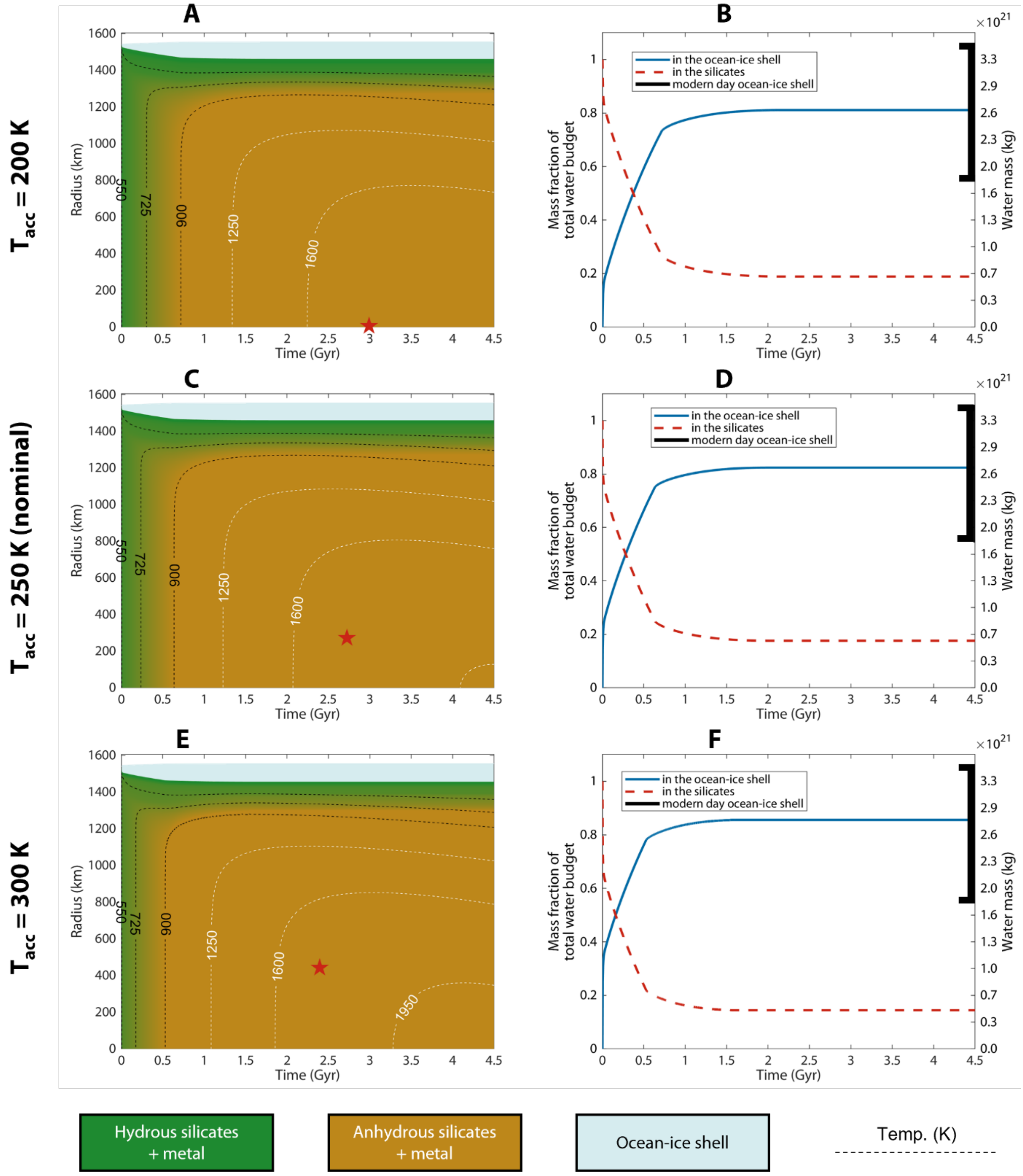

Fig. S4. Model results do not change when varying accretional temperature (T_{acc}) below sub-freezing temperatures. Europa releases enough fluid to form the ocean-ice shell, metallic core formation starts billions of years after accretion, and the outermost silicates remain hydrated. Each row presents a model that varies only by T_{acc} = 200 K (**A**–**B**), 250 K (**C**–**D**), and 300 K (**E**–**F**), which spans the range suggested for Europa according to the gas-starved disc

model (*49*). Silicates start melting around 2.5–3 Gyr after accretion and near the center of Europa, which may inhibit volcanism. The nominal model presented in the main text is in the middle row (T_{acc} = 250 K). All figure labels and symbols are the same as Figure 3 in the main text, though the Fe-FeS liquidus sulfur content are now summarized in Figure S10.

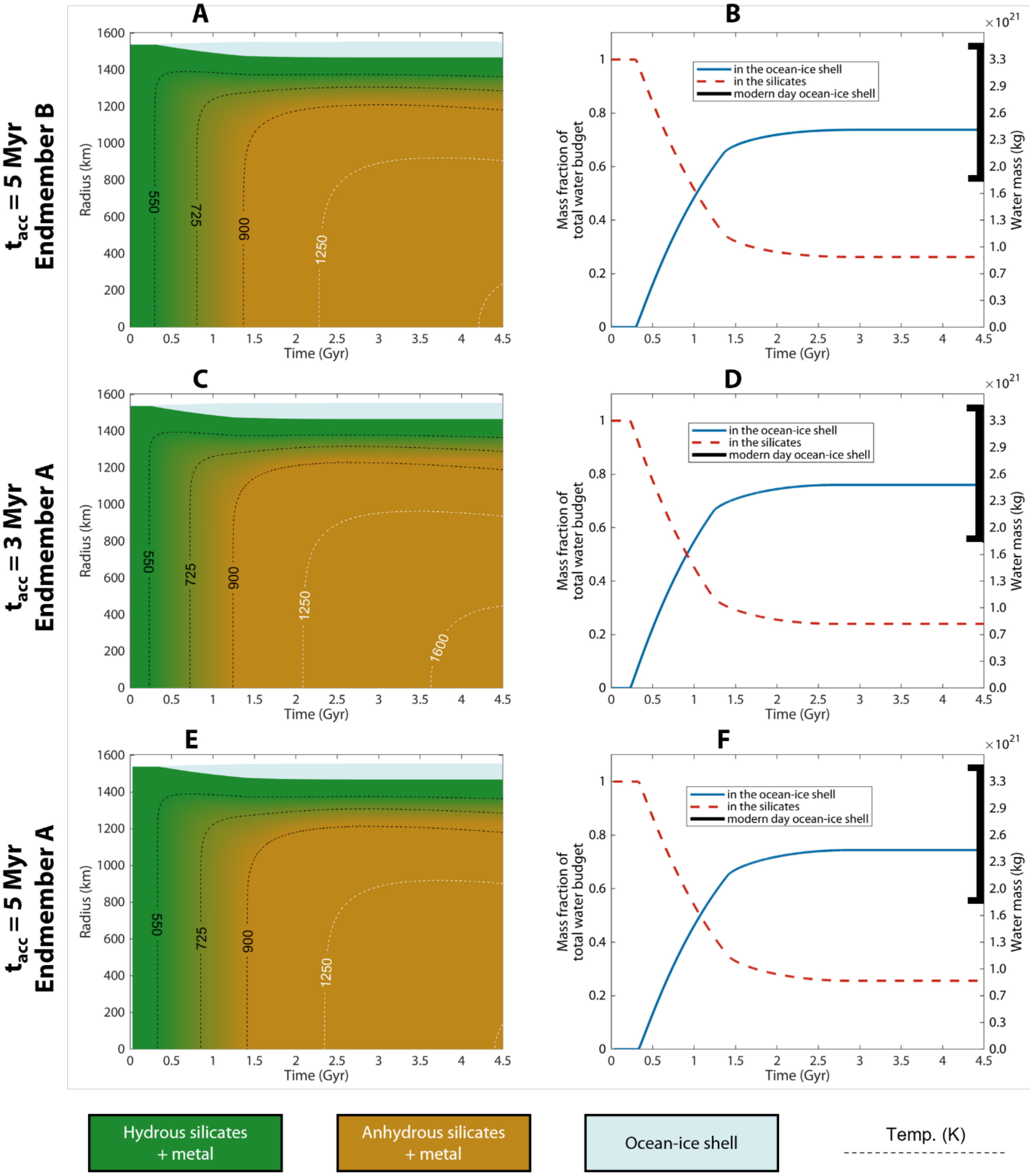

Fig. S5. Model results do not change substantially if Europa accreted ice-rock differentiated or not. Europa releases enough fluid to form the ocean-ice shell, metallic core formation starts billions of years after accretion, and the outermost silicates remain hydrated. The top row shows a model where Europa accreted as a mixture of hydrated silicates and metal (Endmember B) at t_{acc} = 5 Myr (**A**–**B**). The middle and bottom row assumes Europa accreted ice-rock undifferentiated (Endmember A) with t_{acc} = 3 Myr (**C**–**D**) and 5 Myr (**E**–**F**), respectively, and that ice-rock subsequent differentiation results in silicate hydration. The plots start after our

model Europa completes ice-rock differentiation. We assume enough ice was present to result in 6.8 wt.% H_2O by the start of the simulation shown. All figure labels and symbols are the same as Figure 3 in the main text, though the Fe-FeS liquidus sulfur content are now summarized in Figure S10.

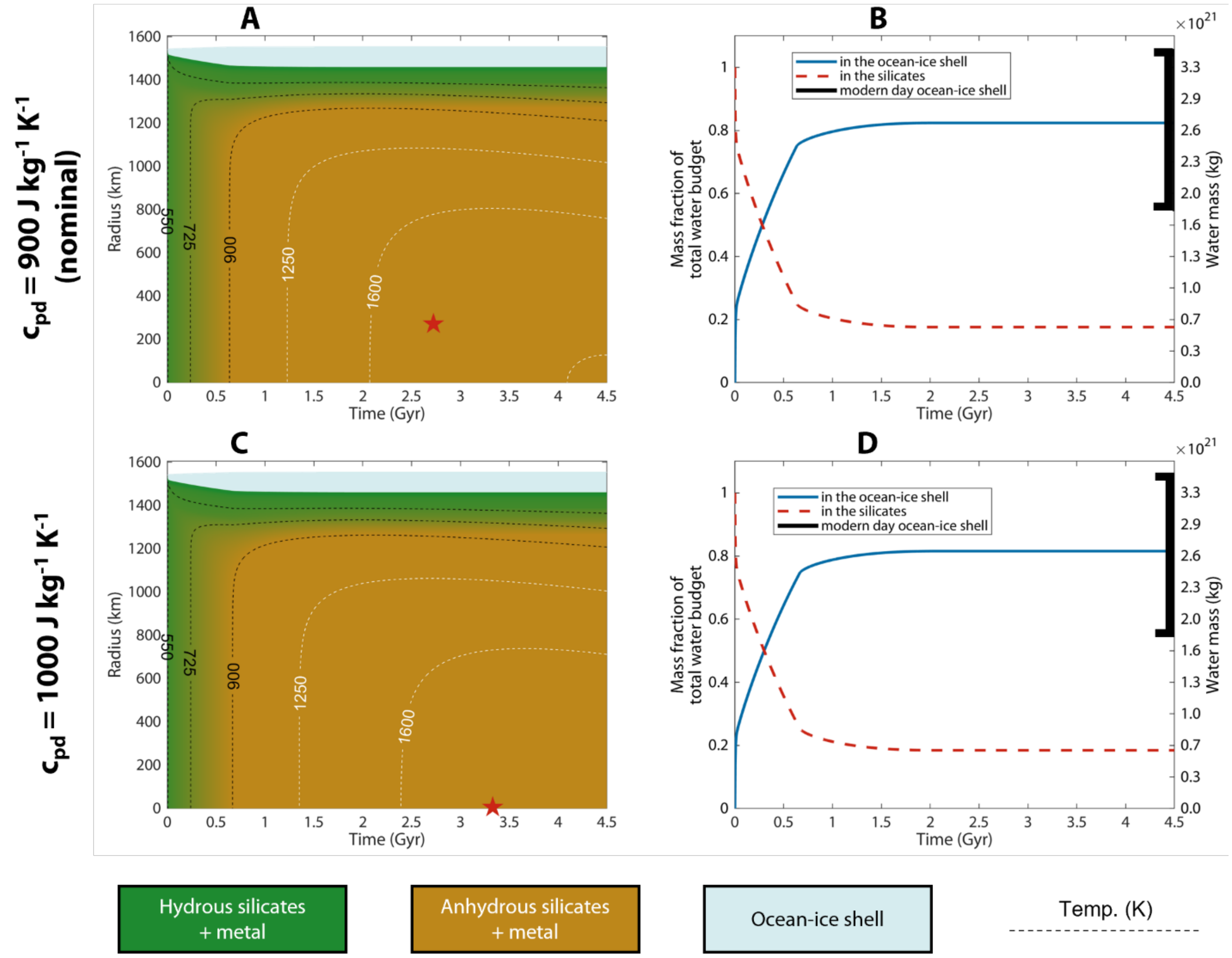

Fig. S6. Model results are not sensitive to the specific heat of anhydrous silicates (c_{pd}) within our explored range. Europa releases enough fluid to form the ocean-ice shell, metallic core formation starts billions of years after accretion, and the outermost silicates remain hydrated. The changes in the timing of metallic core formation and ocean thickness are less than the changes that arise from our uncertainty in t_{acc}, X, and Q_T shown in Figures S3–S5. The top and bottom row show models assuming c_{pd} = 900 J kg^{-1} K^{-1} (nominal) (**A**–**B**) and 1000 J kg^{-1} K^{-1} (**C**–**D**), respectively. All figure labels and symbols are the same as Figure 3 in the main text, though the Fe-FeS liquidus sulfur content are now summarized in Figure S10.

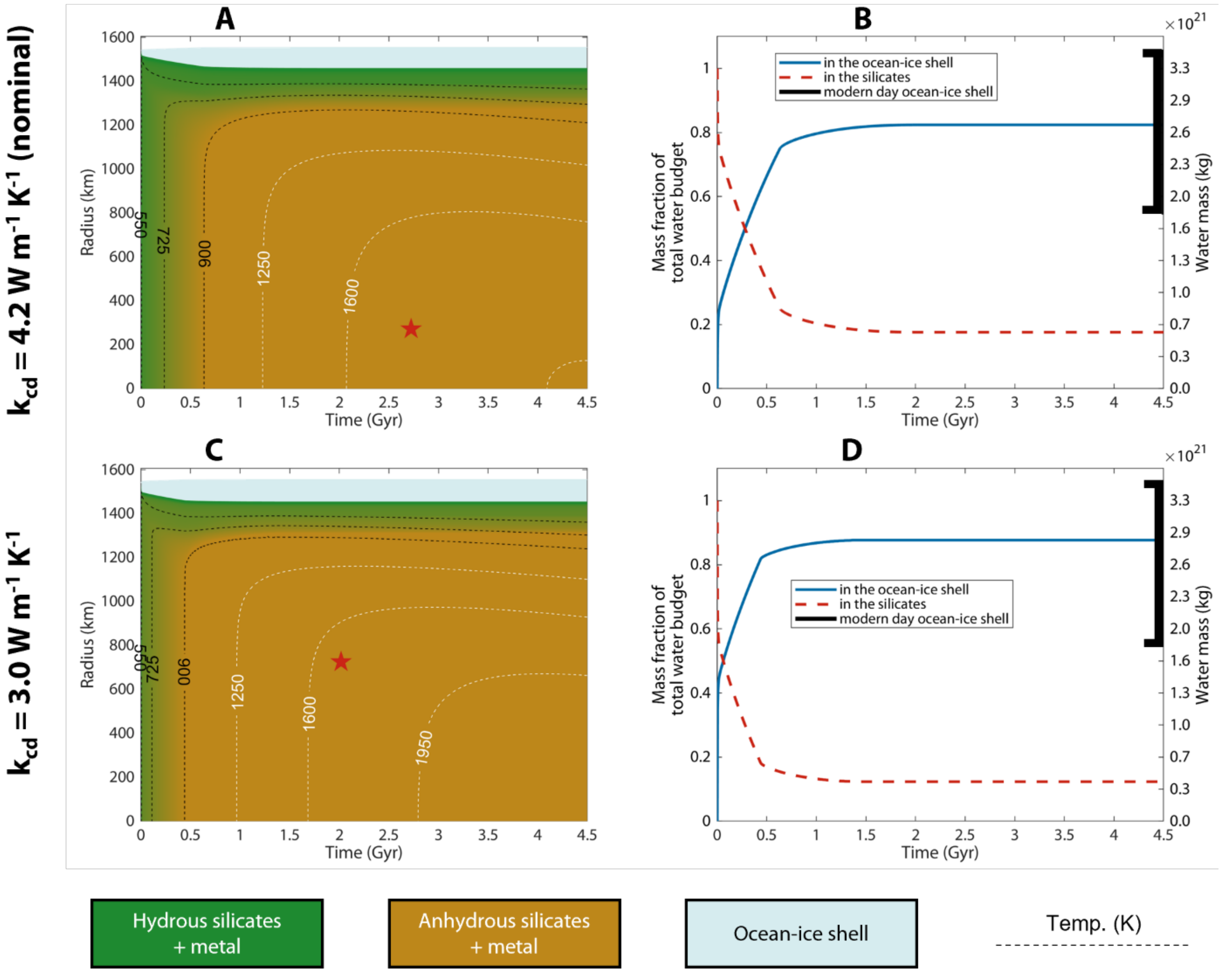

Fig. S7. Model results are not sensitive to the thermal conductivity of anhydrous silicates (k_d) within our explored range. Europa releases enough fluid to form the ocean-ice shell, metallic core formation starts billions of years after accretion, and the outermost silicates remain hydrated. Silicate melting shifts upward by ~500 km when decreasing k_d, but silicate volcanism is still unlikely given melt depth. The changes in the timing of metallic core formation and ocean thickness are less than the changes that arise from our uncertainty in t_{acc}, X, and Q_T shown in Figures S3–S5. The top and bottom row show models assuming k_d = 4.2 W m^{-1} K^{-1} (nominal) (**A**–**B**) and 3.0 W m^{-1} K^{-1} (**C**–**D**), respectively. All figure labels and symbols are the same as Figure 3 in the main text, though the Fe-FeS liquidus sulfur content are now summarized in Figure S10.

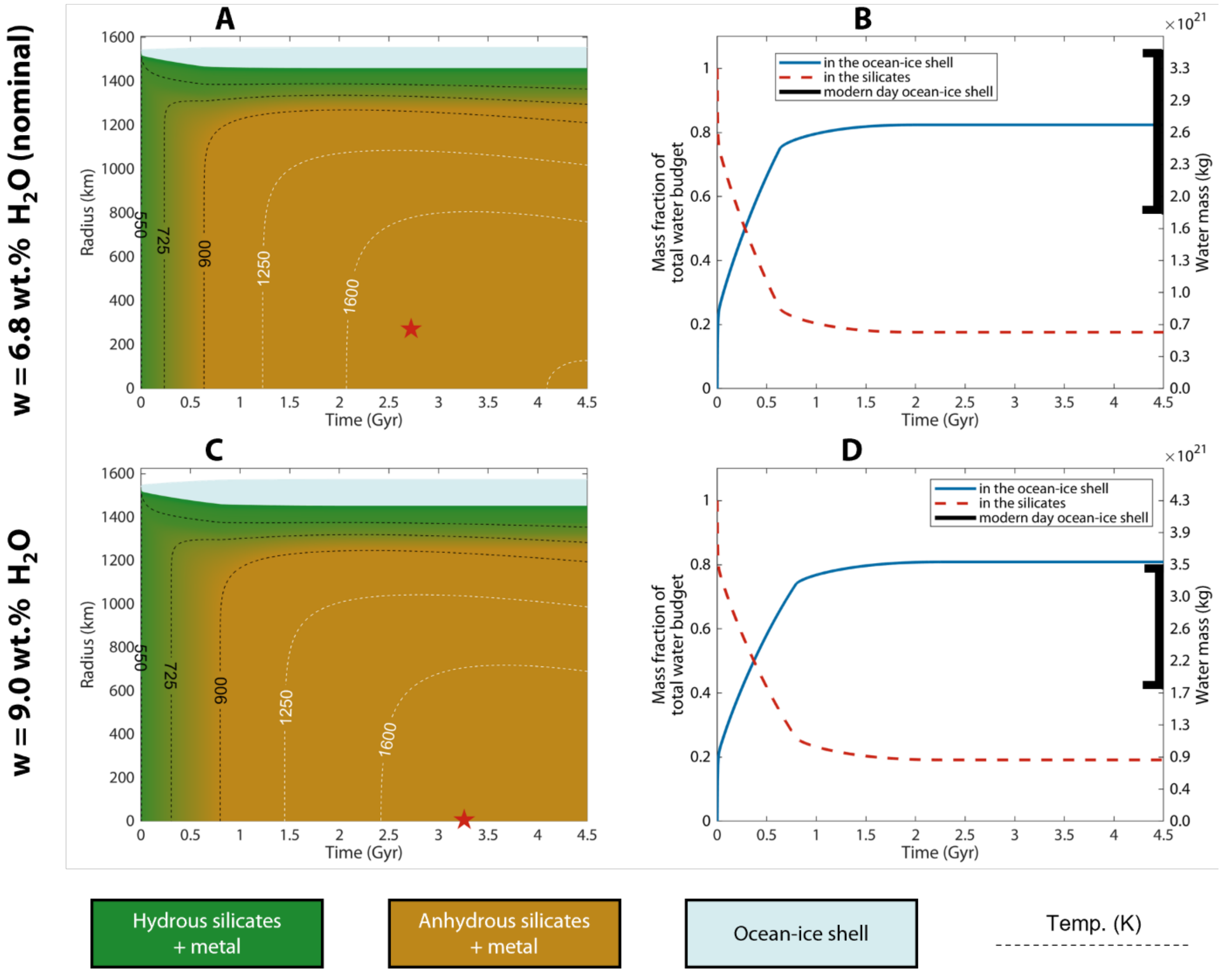

Fig. S8. Too much fluid may be released if Europa's primordial mineral assemblage is > 9 wt.% H_2O. (**A–B**) The nominal model in the top row assumes density and hydration values that are consistent with "Prinn-Fegley 2" (PF2) rock, which is a hypothetical rock composition obtained by energy minimization calculations in the circumjovian nebula (*56*, *57*, *93*). (**C–D**) The bottom row assumes that the hydrated mineral assemblage is ρ_h = 3000 kg m^{-3} and 9 wt.% water, both of which are consistent with the grain density and water content of CM chondrites (*18*, *94*). All other parameters remain the same as the nominal model. All figure labels and symbol are the same as Figure 3 in the main text, though the Fe-FeS liquidus sulfur content are now summarized in Figure S10.

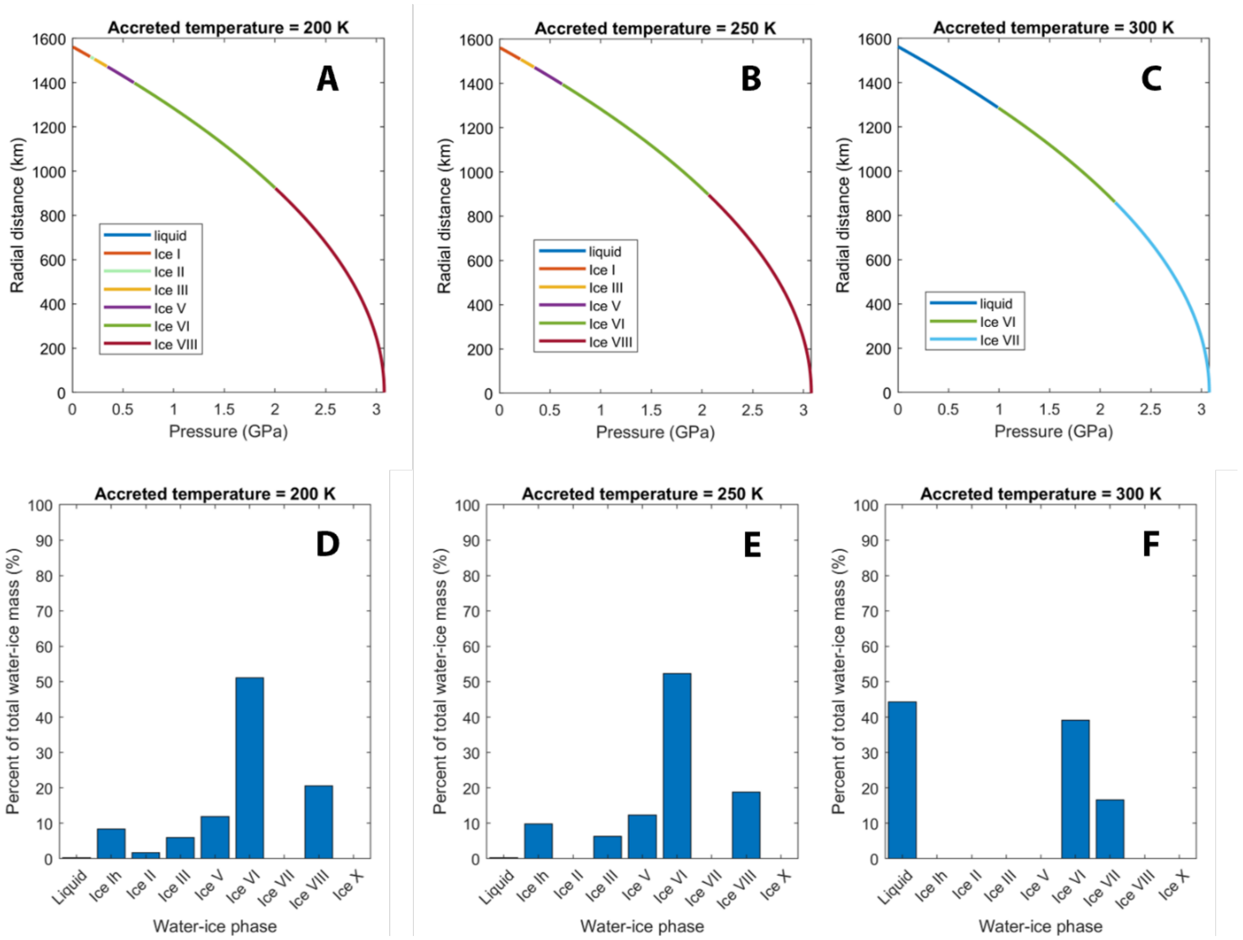

Fig. S9. High-pressure ice stabilities for Europa's ice-rock undifferentiated accreted state used to calculate simulation start times *(t_{init})*. The phases of primordial water-ice at Europa is important for understanding the temperatures required to ice-rock differentiate. The gas-starved disc model suggests that Europa accreted slowly such that Europa's initial temperature profile matched the background disc temperature at Europa's orbital position of about 200–300 K (*49*). We present relative abundances assuming uniform temperatures of t_{acc} = 200 K, 250 K, and 300 K. (**A**–**C**) Relative abundance of high-pressure ices mapped across Europa's radial distance from the center and corresponding pressure. (**D**–**F**) Relative mass abundance of high-pressure ices in Europa.

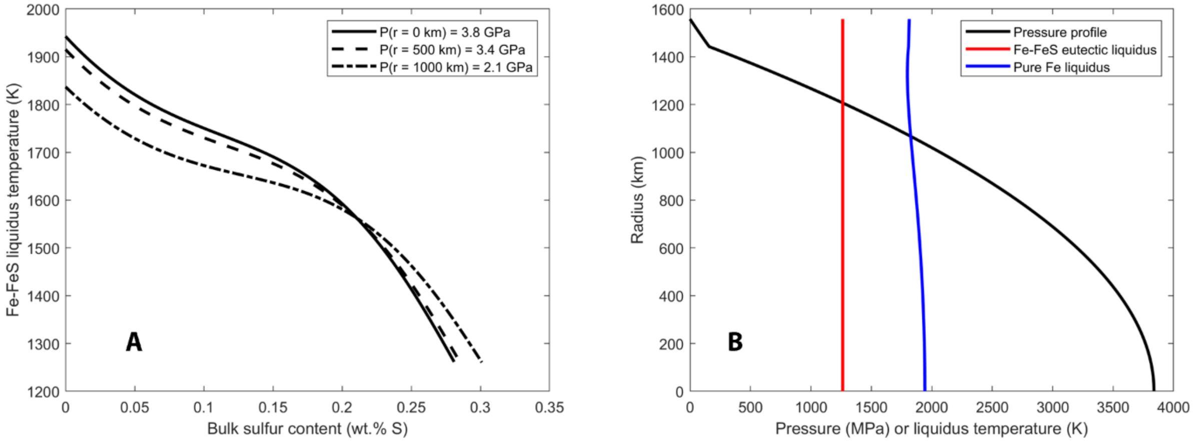

Fig. S10. Fe-FeS liquidus temperatures depend on sulfur abundance and pressure. We calculated liquidus temperatures using Equation 29 from (*46*). (**A**) The Fe-FeS liquidus temperature varies a lot with sulfur content but a little by pressure. The pressures chosen correspond to Europa's central pressure and 500 km intervals from Europa's center. The Fe-FeS eutectic is 1623 ± 25 K for P = 1 bar – 6 GPa, but the liquidus of pure Fe varies by pressure (*46*). (**B**) Europa's pressure profile, Fe-FeS eutectic liquidus, and pure Fe liquidus plotted against Europa's depth. S-poor metallic alloys have lower liquidus temperatures at shallower depths, so metallic core formation may not begin at the moon's center. We need thermal profiles to pinpoint where metallic core formation initiates.

Table S1. Radioactive isotopes used in (*58*) and this work. We include long- (*84*) and short-lived isotopes (*63*, *85*). Values for ^{60}Fe come from (*86*).

Isotope	Concentration (ppb)	Specific heat production (W kg^{-1})	Half-life (yrs)
^{235}U	5.4	568.7×10^{-6}	7.04×10^{8}
^{238}U	19.9	94.65×10^{-6}	4.47×10^{9}
^{40}K	737.9	29.17×10^{-6}	1.28×10^{9}
^{232}Th	38.7	26.38×10^{-6}	1.4×10^{10}
^{26}Al	600	0.355	7.16×10^{6}
^{60}Fe	100	7.0×10^{-2}	1.5×10^{6}
^{53}Mn	25.7	2.7×10^{-2}	3.7×10^{6}

Table S2. Thermophysical model parameters of the nominal case. For assemblages with a mixture of hydrous and anhydrous endmembers, the specific heat capacities are $Xc_{ph} + (1 - X)c_{pd}$ and thermal conductivities are $fk_h + (1 - f)k_d$ where X and f are mass and volume fraction of hydrated silicates, respectively. We assume that Europa accreted material that is compositionally like Prinn-Fegley 2 (PF2) rock which has been obtained by Gibbs free energy minimization calculations on the circumjovian nebula (*93*) and updated with solar abundances (*95*). PF2 rock has been used on models of Titan (*56*) and the Pluto-Charon system (*57*).

Property	Symbol	Endmember	Value	Justification
Specific heat capacity ($J\ kg^{-1}\ K^{-1}$)	c_p	Anhydrous	900	(*91*)
		Hydrous	1000 + 400 (T – 550) / 350 for 550 K < T < 900 K	(*96*)
Thermal conductivity ($W\ m^{-1}\ K^{-1}$)	k	Anhydrous	4.2	(*91*)
		Hydrous	2.7	(*96*)
Density ($kg\ m^{-3}$)	ρ	Anhydrous	3559	Consistent with (*57*)
		Hydrous	3141	(*57*)
Water content (wt.% H_2O)	*w*	Hydrous	6.8	(*57*)
Latent heat of dehydration	*L*	Hydrous	202 $kJ\ kg^{-1}$	(*65*, *91*)

Table S3. Densities and thicknesses of three-layer Europa models. In our work, we use MoI = 0.3547 ± 0.0024 which does not require Europa to behave hydrostatically (*43*). Previous studies assume that Europa is a hydrostatic body by using MoI = 0.346 ± 0.005. While all models presented are geophysically consistent with *Gallileo* radio doppler data, not all models are geologically or geochemically plausible as discussed in the respective studies (*7*, *8*, *10*, *11*, *97*). Interior structure models may be supplemented by geochemical thermodynamics and meteorite data (*7*, *8*). Additionally, the results presented may explore a limited range of plausible density and layer thicknesses.

	Density (kg m^{-3})			Thickness (km)	
Citation	Metallic core	Silicate mantle	Ocean-ice shell	Metallic core	Ocean-ice shell
(*4*)	5150 – 8000	3000 – 3529	900 – 1100	< 782	80 – 170
(*7*)	5500 – 8000	~3250 – 3750	1050	188 – 704	120 – 200
(*8*)	4700 – 5700	3400 – 3520	Variable, not explicitly stated	470 – 640	105 – 150
Model B of (*10*)	4700 – 8000	3500	1000	437 – 702	135 – 137
(*11*)	8000	3425	917 – 1170 (variable)	475	106
(*98*)	Inputs include layer thicknesses (not densities)			450 – 810	106 – 148
Our work	5150 – 8000	3000 – 3550	950 – 1050	258 – 786	55 – 128

REFERENCES AND NOTES

1. C. F. Chyba, C. B. Phillips, Europa as an abode of life. *Orig. Life Evol. Biosph.* **32**, 47–67 (2002).

2. K. P. Hand, C. F. Chyba, J. C. Priscu, R. W. Carlson, K. H. Nealson, "Astrobiology and the Potential for Life on Europa" in *Europa* (The University of Arizona Press, 2017), *The University of Arizona Press*, pp. 589–629.

3. K. P. Hand, C. Sotin, A. Hayes, A. Coustenis, On the habitability and future exploration of ocean worlds. *Space Sci. Rev.* **216**, 95 (2020).

4. J. D. Anderson, G. Schubert, R. A. Jacobson, E. L. Lau, W. B. Moore, W. L. Sjogren, Europa's differentiated internal structure: Inferences from four galileo encounters. *Science* **281**, 2019–2022 (1998).

5. K. K. Khurana, M. G. Kivelson, D. J. Stevenson, G. Schubert, C. T. Russell, R. J. Walker, C. Polanskey, Induced magnetic fields as evidence for subsurface oceans in Europa and Callisto. *Nature* **395**, 777–780 (1998).

6. M. G. Kivelson, K. K. Khurana, C. T. Russell, M. Volwerk, R. J. Walker, C. Zimmer, Galileo magnetometer measurements: A stronger case for a subsurface ocean at Europa. *Science* **289**, 1340–1343 (2000).

7. F. Sohl, T. Spohn, D. Breuer, K. Nagel, Implications from galileo observations on the interior structure and chemistry of the galilean satellites. *Icarus* **157**, 104–119 (2002).

8. O. L. Kuskov, V. A. Kronrod, Internal structure of Europa and Callisto. *Icarus* **177**, 550–569 (2005).

9. V. A. Kronrod, O. L. Kuskov, Chemical differentiation of the Galilean satellites of Jupiter: 4. Isochemical models for the compositions of Io, Europa, and Ganymede. *Geochem. Int.* **44**, 529–546 (2006).

10. G. Schubert, F. Sohl, H. Hussmann, "Interior of Europa" in *Europa* (2009), *The University of*

Arizona Press, pp. 353–367.

11. S. D. Vance, M. P. Panning, S. Stähler, F. Cammarano, B. G. Bills, G. Tobie, S. Kamata, S. Kedar, C. Sotin, W. T. Pike, R. Lorenz, H.-H. Huang, J. M. Jackson, B. Banerdt, Geophysical investigations of habitability in ice-covered ocean worlds. *J. Geophys. Res. Planets* **123**, 180–205 (2018).

12. K. M. Soderlund, K. Kalousová, J. J. Buffo, C. R. Glein, J. C. Goodman, G. Mitri, G. W. Patterson, F. Postberg, M. Rovira-Navarro, T. Rückriemen, J. Saur, B. E. Schmidt, C. Sotin, T. Spohn, G. Tobie, T. Van Hoolst, S. D. Vance, B. Vermeersen, Ice-ocean exchange processes in the Jovian and saturnian satellites. *Space Sci. Rev.* **216**, 80 (2020).

13. T. M. McCollom, Methanogenesis as a potential source of chemical energy for primary biomass production by autotrophic organisms in hydrothermal systems on Europa. *J. Geophys. Res. Planets* **104**, 30729–30742 (1999).

14. S. D. Vance, K. P. Hand, R. T. Pappalardo, Geophysical controls of chemical disequilibria in Europa. *Geophys. Res. Lett.* **43**, 4871–4879 (2016).

15. W. Martin, J. Baross, D. Kelley, M. J. Russell, Hydrothermal vents and the origin of life. *Nat. Rev. Microbiol.* **6**, 805–814 (2008).

16. J. A. M. Leong, E. L. Shock, Thermodynamic constraints on the geochemistry of low-temperature, continental, serpentinization-generated fluids. *Am. J. Sci.* **320**, 185–235 (2020).

17. A. E. Rubin, J. M. Trigo-Rodríguez, H. Huber, J. T. Wasson, Progressive aqueous alteration of CM carbonaceous chondrites. *Geochim. Cosmochim. Acta* **71**, 2361–2382 (2007).

18. A. Garenne, P. Beck, G. Montes-Hernandez, R. Chiriac, F. Toche, E. Quirico, L. Bonal, B. Schmitt, The abundance and stability of “water” in type 1 and 2 carbonaceous chondrites (CI, CM and CR). *Geochim. Cosmochim. Acta* **137**, 93–112 (2014).

19. N. Braukmüller, F. Wombacher, D. C. Hezel, R. Escoube, C. Münker, The chemical composition of carbonaceous chondrites: Implications for volatile element depletion,

complementarity and alteration. *Geochim. Cosmochim. Acta* **239**, 17–48 (2018).

20. B. L. Ehlmann, J. F. Mustard, S. L. Murchie, Geologic setting of serpentine deposits on Mars. *Geophys. Res. Lett.* **37** (2010).

21. H. Lin, J. D. Tarnas, J. F. Mustard, X. Zhang, Y. Wei, W. Wan, F. Klein, J. R. Kellner, Dynamic aperture factor analysis/target transformation (DAFA/TT) for Mg-serpentine and Mg-carbonate mapping on Mars with CRISM near-infrared data. *Icarus* **355**, 114168 (2021).

22. A. J. Dombard, A. M. Sessa, Gravity measurements are key in addressing the habitability of a subsurface ocean in Jupiter's Moon Europa. *Icarus* **325**, 31–38 (2019).

23. M. T. Bland, C. M. Elder, Silicate volcanism on Europa's seafloor and implications for habitability. *Geophys. Res. Lett.* **49**, e2021GL096939 (2022).

24. J. S. Kargel, J. Z. Kaye, J. W. Head, G. M. Marion, R. Sassen, J. K. Crowley, O. P. Ballesteros, S. A. Grant, D. L. Hogenboom, Europa's crust and ocean: Origin, composition, and the prospects for life. *Icarus* **148**, 226–265 (2000).

25. M. Y. Zolotov, E. L. Shock, Composition and stability of salts on the surface of Europa and their oceanic origin. *J. Geophys. Res. Planets* **106**, 32815–32827 (2001).

26. M. Y. Zolotov, E. L. Shock, Energy for biologic sulfate reduction in a hydrothermally formed ocean on Europa. *J. Geophys. Res. Planets* **108**, 10.1029/2002JE001966 (2003).

27. M. Y. Zolotov, E. L. Shock, A model for low-temperature biogeochemistry of sulfur, carbon, and iron on Europa. *J. Geophys. Res. Planets* **109**, 10.1029/2003JE002194 (2004).

28. W. B. McKinnon, M. E. Zolensky, Sulfate content of Europa's ocean and shell: Evolutionary considerations and some geological and astrobiological implications. *Astrobiology* **3**, 879–897 (2003).

29. T. B. McCord, G. B. Hansen, F. P. Fanale, R. W. Carlson, D. L. Matson, T. V. Johnson, W.

D. Smythe, J. K. Crowley, P. D. Martin, A. Ocampo, C. A. Hibbitts, J. C. Granahan, Salts on Europa's surface detected by Galileo's near infrared mapping spectrometer. The NIMS Team. *Science* **280**, 1242–1245 (1998).

30. T. B. McCord, G. B. Hansen, D. L. Matson, T. V. Johnson, J. K. Crowley, F. P. Fanale, R. W. Carlson, W. D. Smythe, P. D. Martin, C. A. Hibbitts, J. C. Granahan, A. Ocampo, Hydrated salt minerals on Europa’s surface from the Galileo near-infrared mapping spectrometer (NIMS) investigation. *J. Geophys. Res. Planets* **104**, 11827–11851 (1999).

31. T. B. McCord, G. Teeter, G. B. Hansen, M. T. Sieger, T. M. Orlando, Brines exposed to Europa surface conditions. *J. Geophys. Res. Planets* **107**, 4-1-4–6 (2002).

32. T. M. Orlando, T. B. McCord, G. A. Grieves, The chemical nature of Europa surface material and the relation to a subsurface ocean. *Icarus* **177**, 528–533 (2005).

33. R. W. Carlson, R. E. Johnson, M. S. Anderson, Sulfuric acid on Europa and the radiolytic sulfur cycle. *Science* **286**, 97–99 (1999).

34. R. W. Carlson, M. S. Anderson, R. E. Johnson, M. B. Schulman, A. H. Yavrouian, Sulfuric acid production on Europa: The radiolysis of sulfur in water ice. *Icarus* **157**, 456–463 (2002).

35. R. W. Carlson, M. S. Anderson, R. Mehlman, R. E. Johnson, Distribution of hydrate on Europa: Further evidence for sulfuric acid hydrate. *Icarus* **177**, 461–471 (2005).

36. A. Néri, F. Guyot, B. Reynard, C. Sotin, A carbonaceous chondrite and cometary origin for icy moons of Jupiter and Saturn. *Earth Planet. Sci. Lett.* **530**, 115920 (2020).

37. G. A. Ransford, A. A. Finnerty, K. D. Collerson, Europa’s petrological thermal history. *Nature* **289**, 21–24 (1981).

38. M. Melwani Daswani, S. D. Vance, M. J. Mayne, C. R. Glein, A metamorphic origin for Europa's ocean. *Geophys. Res. Lett.* **48**, e2021GL094143, (2021).

39. S. K. Trumbo, M. E. Brown, K. P. Hand, Sodium chloride on the surface of Europa. *Sci. Adv.* **5**, eaaw7123 (2019).

40. S. K. Trumbo, T. M. Becker, M. E. Brown, W. T. P. Denman, P. Molyneux, A. Hendrix, K. D. Retherford, L. Roth, J. Alday, A new UV spectral feature on Europa: Confirmation of NaCl in leading-hemisphere chaos terrain. *Planet. Sci. J.* **3**, 27 (2022).

41. M. E. Brown, W. T. P. Denman, S. K. Trumbo, The mid-UV spectrum of irradiated NaCl at Europa-like conditions. *Planet. Sci. J.* **3**, 28 (2022).

42. J. J. Buffo, B. E. Schmidt, C. Huber, C. C. Walker, Entrainment and dynamics of ocean-derived impurities within Europa's ice shell. *J. Geophys. Res. Planets* **125**, e2020JE006394 (2020).

43. L. Gomez Casajus, M. Zannoni, D. Modenini, P. Tortora, F. Nimmo, T. Van Hoolst, D. Buccino, K. Oudrhiri, Updated Europa gravity field and interior structure from a reanalysis of Galileo tracking data. *Icarus* **358**, 114187 (2021).

44. B. J. Travis, J. Palguta, G. Schubert, A whole-moon thermal history model of Europa: Impact of hydrothermal circulation and salt transport. *Icarus* **218**, 1007–1019 (2012).

45. M. Běhounková, G. Tobie, G. Choblet, M. Kervazo, M. Melwani Daswani, C. Dumoulin, S. D. Vance, Tidally induced magmatic pulses on the oceanic floor of Jupiter's moon Europa. *Geophys. Res. Lett.* **48**, e2020GL090077 (2021).

46. A. S. Buono, D. Walker, The Fe-rich liquidus in the Fe–FeS system from 1bar to 10GPa. *Geochim. Cosmochim. Acta* **75**, 2072–2087 (2011).

47. J. Kimura, T. Nakagawa, K. Kurita, Size and compositional constraints of Ganymede’s metallic core for driving an active dynamo. *Icarus* **202**, 216–224 (2009).

48. R. A. Fischer, Y. Nakajima, A. J. Campbell, D. J. Frost, D. Harries, F. Langenhorst, N. Miyajima, K. Pollok, D. C. Rubie, High pressure metal–silicate partitioning of Ni, Co, V, Cr, Si, and O. *Geochim. Cosmochim. Acta* **167**, 177–194 (2015).

49. R. M. Canup, W. R. Ward, Formation of the Galilean satellites: Conditions of accretion. *Astron. J.* **124**, 3404–3423 (2002).

50. A. N. Dunaeva, D. V. Antsyshkin, O. L. Kuskov, Phase diagram of H2O: Thermodynamic functions of the phase transitions of high-pressure ices. *Sol. Syst. Res.* **44**, 202–222 (2010).

51. S. W. Squyres, R. T. Reynolds, P. M. Cassen, S. J. Peale, Liquid water and active resurfacing on Europa. *Nature* **301**, 225–226 (1983).

52. R. E. Grimm, H. Y. McSween Jr., Heliocentric zoning of the asteroid belt by aluminum-26 heating. *Science* **259**, 653–655 (1993).

53. R. M. Canup, W. R. Ward, A common mass scaling for satellite systems of gaseous planets. *Nature* **441**, 834–839 (2006).

54. T. S. Kruijer, C. Burkhardt, G. Budde, T. Kleine, Age of Jupiter inferred from the distinct genetics and formation times of meteorites. *Proc. Natl. Acad. Sci. U.S.A.* **114**, 6712–6716 (2017).

55. E. J. Gaidos, K. H. Nealson, J. L. Kirschvink, Life in ice-covered oceans. *Science* **284**, 1631–1633 (1999).

56. W. B. McKinnon, M. T. Bland, Core evolution in icy satellites and Kuiper belt objects, 42nd Lunar and Planetary Science Conference. LPI Contribution No. 1608, p. 2768, (2011).

57. W. B. McKinnon, S. A. Stern, H. A. Weaver, F. Nimmo, C. J. Bierson, W. M. Grundy, J. C. Cook, D. P. Cruikshank, A. H. Parker, J. M. Moore, J. R. Spencer, L. A. Young, C. B. Olkin, K. E. Smith, Origin of the Pluto–Charon system: Constraints from the New Horizons flyby. *Icarus* **287**, 2–11 (2017).

58. C. J. Bierson, F. Nimmo, Using the density of Kuiper belt objects to constrain their composition and formation history. *Icarus* **326**, 10–17 (2019).

59. G. W. Ojakangas, D. J. Stevenson, Thermal state of an ice shell on Europa. *Icarus* **81**, 220–

241 (1989).

60. C. Sotin, G. Tobie, J. Wahr, W. B. McKinnon, "Tides and Tidal Heating on Europa" in *Europa* (2009), *The University of Arizona Press*.

61. W. B. Moore, H. Hussmann, "Thermal Evolution of Europa's Silicate Interior" in *Europa* (University of Arizona Press, 2017), *The University of Arizona Press*, pp. 369–380.

62. H. Hussmann, T. Spohn, Thermal-orbital evolution of Io and Europa. *Icarus* **171**, 391–410 (2004).

63. J. Castillo-Rogez, T. V. Johnson, M. H. Lee, N. J. Turner, D. L. Matson, J. Lunine, 26Al decay: Heat production and a revised age for Iapetus. *Icarus* **204**, 658–662 (2009).

64. M. Neveu, S. J. Desch, J. C. Castillo-Rogez, Aqueous geochemistry in icy world interiors: Equilibrium fluid, rock, and gas compositions, and fate of antifreezes and radionuclides. *Geochim. Cosmochim. Acta* **212**, 324–371 (2017).

65. J. N. Weber, R. T. Greer, Dehydration of serpentine: Heat of reaction and reaction kinetics at PH2O=1 ATM. *Am. Mineral.* **50**, 450–464 (1965).

66. R. F. Katz, M. Spiegelman, C. H. Langmuir, A new parameterization of hydrous mantle melting. *Geochem. Geophys. Geosyst.* **4**, 10.1029/2002GC000433 (2003).

67. R. Dasgupta, A. Buono, G. Whelan, D. Walker, High-pressure melting relations in Fe–C–S systems: Implications for formation, evolution, and structure of metallic cores in planetary bodies. *Geochim. Cosmochim. Acta* **73**, 6678–6691 (2009).

68. J. S. Kargel, Brine volcanism and the interior structures of asteroids and icy satellites. *Icarus* **94**, 368–390 (1991).

69. C. J. Bierson, G. Steinbrügge, Tidal heating did not dry out Io and Europa. *Planet. Sci. J.* **2**, 89 (2021).

70. R. Greeley, C. F. Chyba, J. W. Head III, T. B. McCord, W. B. McKinnon, R. T. Pappalardo,

P. Figueredo, "Geology of Europa" in *Jupiter The Planet, Satellites, and Magnetosphere* (2004), pp. 329–362.

71. S. Vance, J. Harnmeijer, J. Kimura, H. Hussmann, B. deMartin, J. M. Brown, Hydrothermal systems in small ocean planets. *Astrobiology* **7**, 987–1005 (2007).

72. M. Y. Zolotov, J. S. Kargel, "On the Chemical Composition of Europa's Icy Shell, Ocean, and Underlying Rocks" in *Europa* (2009), *The University of Arizona Press*, pp. 431–457.

73. G. Schubert, J. D. Anderson, T. Spohn, W. B. McKinnon, Interior Composition, Structure, and Dynamics of the Galilean Satellites. *in Jupiter The planet, satellites and magnetosphere* (2004), pp. 281–306.

74. J. I. Lunine, D. J. Stevenson, Formation of the galilean satellites in a gaseous nebula. *Icarus* **52**, 14–39 (1982).

75. I. Mosqueira, P. R. Estrada, Formation of the regular satellites of giant planets in an extended gaseous nebula II: Satellite migration and survival. *Icarus* **163**, 232–255 (2003).

76. P. R. Estrada, I. Mosqueira, A gas-poor planetesimal capture model for the formation of giant planet satellite systems. *Icarus* **181**, 486–509 (2006).

77. T. Ronnet, O. Mousis, P. Vernazza, Pebble accretion at the origin of water in Europa. *Astrophys. J.* **845**, 92 (2017).

78. C. J. Bierson, F. Nimmo, Explaining the Galilean Satellites' Density Gradient by Hydrodynamic Escape. *Astrophys. J. Lett.* **897**, L43 (2020).

79. O. Mousis, A. Schneeberger, J. I. Lunine, C. R. Glein, A. Bouquet, S. D. Vance, Early stages of galilean moon formation in a water-depleted environment. *Astrophys. J. Lett.* **944**, L37 (2023).

80. G. Madeira, A. Izidoro, S. M. Giuliatti Winter, Building the Galilean moons system via pebble accretion and migration: A primordial resonant chain. *Mon. Notices Royal Astron.*

Soc. **504**, 1854–1872 (2021).

81. R. M. Canup, W. R. Ward, "Origin of Europa and the Galilean Satellites" in *Europa* (2009), *The University of Arizona Press*, pp. 59–84.

82. C. A. Dwyer, F. Nimmo, M. Ogihara, S. Ida, The influence of imperfect accretion and radial mixing on ice:rock ratios in the Galilean satellites. *Icarus* **225**, 390–402 (2013).

83. R. E. Johnson, The Magnetospheric Plasma-driven Evolution of Satellite Atmospheres. *Astrophys. J.* **609**, L99–L102 (2004).

84. G. Robuchon, F. Nimmo, Thermal evolution of Pluto and implications for surface tectonics and a subsurface ocean. *Icarus* **216**, 426–439 (2011).

85. J. C. Castillo-Rogez, D. L. Matson, C. Sotin, T. V. Johnson, J. I. Lunine, P. C. Thomas, Iapetus' geophysics: Rotation rate, shape, and equatorial ridge. *Icarus* **190**, 179–202 (2007).

86. H. Tang, N. Dauphas, Abundance, distribution, and origin of 60Fe in the solar protoplanetary disk. *Earth Planet. Sci. Lett.* **359-360**, 248–263 (2012).

87. C. J. Bierson, F. Nimmo, W. B. McKinnon, Implications of the observed Pluto–Charon density contrast. *Icarus* **309**, 207–219 (2018).

88. G. K. Bhatia, S. Sahijpal, The early thermal evolution of Mars. *Meteorit. Planet. Sci.* **51**, 138–154 (2016).

89. M. Ogihara, S. Ida, N-body simulations of satellite formation around giant planets: Origin of orbital configuration of the galilean moons. *Astrophys. J.* **753**, 60 (2012).

90. J. C. Castillo-Rogez, J. I. Lunine, Evolution of Titan's rocky core constrained by Cassini observations. *Geophys. Res. Lett.* **37**, 10.1029/2010GL044398 (2010).

91. K. Nakajima, H. Hasegawa, S. Khumkoa, S. Mizoguchi, Effect of a catalyst on heterogeneous nucleation in pure and Fe-Ni alloys. *Metall. Mater. Trans. B* **34**, 539–547 (2003).

92. R. G. Prinn, B. Fegley Jr., Kinetic inhibition of CO and N2 reduction in circumplanetary nebulae - Implications for satellite composition. *Astrophys. J.* **249**, 308–317 (1981).

93. R. J. Macke, G. J. Consolmagno, D. T. Britt, Density, porosity, and magnetic susceptibility of carbonaceous chondrites. *Meteorit. Planet. Sci.* **46**, 1842–1862 (2011).

94. K. Lodders, Solar system abundances and condensation temperatures of the elements. *Astrophys. J.* **591**, 1220–1247 (2003).

95. M. Osako, A. Yoneda, E. Ito, Thermal diffusivity, thermal conductivity and heat capacity of serpentine (antigorite) under high pressure. *Phys. Earth Planet. In.* **183**, 229–233 (2010).

96. J. D. Anderson, W. L. Scholgren, G. Schubert, Galileo gravity results and the internal structure of Io. *Science* **272**, 709–712 (1996).

97. S. M. Howell, The likely thickness of Europa’s icy shell. *Planet. Sci. J.* **2**, 129 (2021).
